# Persistent prostaglandin E2 upregulation and hormonal multi-resistance: A hypothesis for long COVID

**DOI:** 10.1016/j.bbrep.2026.102518

**Published:** 2026-03-10

**Authors:** Vjera Ramer, Gert A. van Montfrans

**Affiliations:** a Independent Researcher, Amsterdam, the Netherlands; b Academic Medical Centre, University of Amsterdam, Department of Internal Medicine, the Netherlands

## Abstract

An explanation for long COVID (LC) and its many symptoms remains elusive, and no unifying upstream mediator of its pathophysiology has yet been identified. Viral infections, including COVID-19, upregulate Prostaglandin E2 (PGE2), - the principal lipid mediator of inflammation. PGE2 has broad paracrine effects, including modulation of autoantibodies, nervous systems, blood pressure, glucose levels, and apoptosis. Upregulated, PGE2 may drive multiple pathological processes through its four receptors: EP1-EP4. Through EP3, PGE2 stimulates characteristic viral sickness symptoms. Both severe and mild disease may precede LC, possibly reflecting either excessive or insufficient EP3 activity, which we term “EP3 up” and “EP3 down”, respectively. In individuals with “EP3 up” or “EP3 down” states, elevated PGE2 levels may disrupt homeostasis. Diverse physical and mental stressors upregulate PGE2. Data suggest that multiple hormones and agents stimulate PGE2 production, while PGE2 reportedly antagonizes these agents, possibly functioning as a homeostatic limiter. Sufficiently elevated PGE2, “PGE2 dominance”, may interfere with their activity through inhibiting their release, overlapping subcellular signaling pathways or by stimulating phosphorylation and autoantibodies. Homeostatic elevations of PGE2-stimulating hormones, together with other PGE2-raising factors, may promote resistance to these hormones, resulting in persistent PGE2 upregulation. Literature suggests that PGE2 overactivity, mediated by imbalanced EP receptor signaling, aligns with the diverse symptomatology of LC. Based on our literature analysis, we propose that persistent upregulation of PGE2 plays a pivotal role in the development of LC. Consequently, validation of elements of this framework may help guide exploration of therapeutic strategies targeting PGE2 pathways or EP receptor regulation.

## Highlights

• *Persistent Prostaglandin E2 (PGE2) elevation may explain long COVID symptomatology*.
• *PGE2, the principal lipid mediator of inflammation, is raised by viral infections*.
• *PGE2 stimulates autoantibodies to receptors, possibly hindering ligand binding*.
• *PGE2 reportedly antagonizes hormones and agents that stimulate PGE2 production*.
• *"PGE2-dominance" could cause resistances that fuel its persistent upregulation*.

## Graphical abstract

**Figure:**
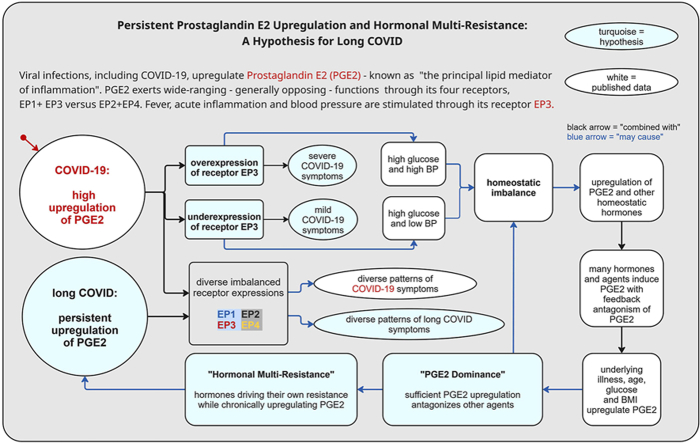

## 1 Introduction

Long COVID (LC), or post-COVID-19 syndrome, is a debilitating condition affecting at least 10% of individuals following either severe acute or mild SARS-CoV-2 infection. More than 200 symptoms and comorbidities have been documented, forming distinct but overlapping clinical clusters. Multiple hypotheses have been proposed for its pathogenesis [1]. However, none provides a unifying molecular framework that accounts for the wide-ranging abnormalities observed across different organs. Reported mechanisms include persistent post-acute inflammation, autoimmune responses, thrombosis, autonomic and mitochondrial dysfunction [1], yet the molecular pathways linking these processes to SARS-CoV-2 infection remain unclear.

In this article, we propose an integrative mechanistic hypothesis that connects LC-associated symptoms, comorbidities, and biological pathways through dysregulation of prostaglandin E2 (PGE2), a central inflammation mediator, and its four cognate receptors, EP1- EP4 [2]. PGE2 synthesis is upregulated in response to both psychological [3] and physiological stressors, including tissue injury and infectious disease [2]. Together with other prostanoids PGE2 mediates the systemic manifestations of illness collectively described as the “sickness syndrome” [2]. Signaling through its EP3 receptor, PGE2 induces hallmark features of viral infection, including headache, fever, and inflammation of the respiratory tract [4]. Circulating PGE2 levels have been reported to be markedly elevated in COVID-19 patients [5]. We hypothesize that elevated PGE2 concentrations may contribute to differential disease severity, with high or low EP3 expression favoring severe or mild COVID-19, respectively. More broadly, sustained PGE2 signaling through its four receptors may cause diverse patterns of organ injury, consistent with pathological findings in COVID-19 [6]. Under physiological conditions, PGE2 levels decline following resolution of the initiating stressor. In contrast, some Long COVID studies mention persistent elevation of PGE2 [7,8] along with increased levels of downstream prostanoids induced by PGE2 signaling, including TXA2 and PGD2 [9]. We therefore hypothesize that prolonged PGE2 dysregulation plays a central role in the post-acute sequelae of COVID-19, analogous to its role during acute infection [10]. Supporting this view, studies conducted outside the specific context of LC - demonstrate that PGE2 and its EP1 - EP4 receptors are mechanistically implicated in many of the individual symptoms, comorbidities, and biological processes associated with LC (see Appendix A, Multimedia component 1: The Wide-ranging Functions of Prostaglandin E2). Expression levels of EP receptors can vary substantially among individuals (see section 3). Imbalanced receptor expression may underlie distinct clinical phenotypes observed in both COVID-19 [6] and LC [11]. We propose a molecular mechanism in which sustained PGE2 upregulation persists after viral clearance, potentially driven by dysregulated EP receptor expression and impaired restoration of homeostatic signaling. In this contribution, we synthesize available evidence to support a “PGE2 hypothesis for Long COVID” (see section 4.4).

## 2 Methodology

This hypothesis-driven narrative review surveyed research on PGE2 and EP receptors in Long COVID and related post-viral syndromes. We searched Scopus, PubMed, Web of Science, and Google Scholar (1980–2025) using combinations of Long COVID terms (“Long COVID” OR “PASC”) OR PGE2/EP receptor terms (“PGE2” OR “COX-2” OR “EP1–EP4” OR “cAMP”), hormones and mediators (“Insulin” OR “Leptin” OR “Dopamine” OR “Cytokines” OR “Autoantibodies”), mechanistic keywords (“Viral persistence” OR “Autoimmunity” OR “Mitochondrial dysfunction”), therapies tested for LC, and PGE2-targeted potential interventions. Long-term multisystem symptom persistence has been documented in large international cohorts [12], alongside emerging mechanistic insights into neurological sequelae of post-acute COVID-19 [13]. For each symptom cluster (systemic, cardiovascular, endocrine, musculoskeletal, HEENT (Head, Eyes, Ears, Nose, Throat), immunologic, pulmonary, neurological, psychoneurological), PGE2-related terms were combined with keywords, symptoms reported as most frequent [12,13]. These searches were supplemented by literature on acute COVID-19 or related post-viral syndromes, myalgic encephalomyelitis/chronic fatigue syndrome (ME/CFS), where mechanistic overlap with long COVID was plausible.

Given the heterogeneity of Long COVID, additional targeted searches were conducted to explore mechanistic links between PGE2 functions and individual symptom domains commonly reported in Long COVID.

Additionally, mechanistic inferences were often derived from studies outside the specific context of long COVID, reflecting the limited activity of direct evidence. These limitations were addressed by emphasizing convergent biological pathways rather than single-study findings.

We selected articles presenting experimental, mechanistic, or review data on PGE2 and its receptors, published between 1980 and 2025. Worth noting, the 1980s and 90s saw major advances in prostaglandin research [14]. Data were extracted qualitatively, focusing on EP receptor and PGE2 findings and their clinical associations. Evidence was synthesized thematically to construct a mechanistic framework linking PGE2 dysregulation to Long COVID symptomatology, supplemented by studies from acute COVID-19 and related post-viral syndromes. In total, 2608 papers were reviewed, including clinical reviews, trials, case reports, and symptom-focused research, predominantly mechanistic studies.

A hypothesis explaining the pathology of long COVID and its diverse symptoms requires extensive referencing. Supplementary data in Appendix A. provide extended information.

• Multimedia component 1: The wide-ranging functions of PGE2
• Multimedia component 2: Hypothetical models of hormonal resistance to AVP, insulin, leptin, FGF23, IFN-gamma and serotonin
• Multimedia component 3: Four LC subphenotypes and clinical consequences of a validated hypothesis
• Multimedia component 4: PGE2 - DA interactions, relevant for olfactory dysfunction, PEM, and POTS?

In the following sections, results from our literature study are broadly categorized: Prostaglandins and their receptors, distribution, functions, their role in COVID-19, the “PGE2 hypothesis”, the potential central role of receptor EP3 in LC and the concept of “hormonal multi-resistance”, four observed LC subphenotypes associated with hypothetical EP receptor expression-patterns, and emerging therapeutic options.

## 3 Prostaglandin E2 and its four EP receptors: Distribution and functions

Prostaglandins, or prostanoids, are paracrine hormones belonging to the eicosanoid family. Eicosanoids are defined as bioactive lipids derived from 20-carbon polyunsaturated fatty acids (PUFAs), primarily from omega-6 arachidonic acid (AA). The rate-limiting enzymes for PGE2 synthesis are cyclooxygenase-1 (COX-1) and cyclooxygenase-2 (COX-2), the latter predominantly under pathological conditions [15].

Prostaglandins can be synthesized in nearly all human cells [16]. PGE2 and prostanoid receptors have also been detected in plants [17] and certain microorganisms [18].

PGE2 exerts its effects through four E-type prostanoid receptors - EP1, EP2, EP3, and EP4 - which are distributed differently among individuals and tissues [15]. Receptors EP3 and EP4 are located in the plasma membranes [15] and nuclei [19] of most cells. EP receptors are also present in mitochondrial membranes [20]. While speculative, these observations could be interpreted as indicative of an ancient evolutionary origin, with potential roles at the most upstream level of biochemical signaling.

PGE2 binds to the four EP receptors with differing affinities, showing the highest affinity for EP4, followed by EP3, then EP2, and the lowest affinity for EP1[21]. EP receptors are G-protein-coupled receptors (GPCRs) that typically couple to different G-proteins: Gi, Gs, and Gq [21]. EP3- and EP1-associated signaling, involving Gi and Gq coupling, often exerts effects that counteract those mediated by predominantly Gs-coupled EP2 and EP4, for example, vasoconstriction versus vasodilation [22]. This corresponds to the actions of the key second messenger cyclic AMP (cAMP), which is upregulated by the Gs pathway but generally downregulated by the Gi pathway [22] (see Table 1 in section 3.1). It should be noted that EP3 exists in splice variants, a minority of which can increase cAMP [21].

### 3.1 EP receptor expression and interaction with AA/COX-2/PGE2 levels

EP4 activation by PGE2 is predominantly linked to anti-inflammatory effects [23] but prolonged stimulation by PGE2 promotes EP4 receptor desensitization, and alternative signaling pathways can support inflammatory responses such as β-arrestin-biased signaling [24], depending on the context [21]. Excessive PGE2 signaling through EP4 and the other EP receptors can have detrimental effects, such as autoimmune reactions [25] and apoptosis [26].

Binding to EP2/EP4, PGE2 can upregulate COX-2 [27], establishing a self-enhancing loop, since COX-2 is required for the synthesis of PGE2 itself and other “prostaglandins of the 2-series,” including thromboxane A2 (TXA2), prostaglandin D2 (PGD2), and prostacyclin (PGI2) [15]. This PGE2-EP2-COX2 feed-forward loop may be counterbalanced or stimulated by the following mechanisms.

• COX-2 is a limiting enzyme for PGE2 synthesis [15].
• COX-2 upregulates EP1[21].
• EP1 upregulation is linked to COX-2 downregulation [28].
• This process is disrupted following binding of AA to COX-2 [29]
• Elevated levels of AA have been observed in COVID-19 [30] and long COVID [31].
• PGE2 is derived from fatty acid AA [15].

Thus, EP1 might function as an indirect limiter on PGE2-production, unless AA levels are too high upregulated. EP receptor expression and mRNA expression depends on genetic factors [15] but may be upregulated or downregulated under certain conditions [29,32,33]. High COX-2/PGE2 levels are reported to upregulate the mRNA and expression of EP2 and (often) EP4 [34]. Expression and mRNA of EP3 are typically downregulated by high PGE2 levels [35]. An overview of these interactions is presented in Table 1.

**Table 1:** EP receptors, their expression, signaling pathways and interaction with COX2/PGE2 levels.

EP receptor	Primary G-protein coupling	Regulation of cAMP or Ca2+ by EP receptor subtypes	Effects of COX-2/PGE2 upregulation on EP mRNA expression	Reciprocal regulation of EP receptors and COX-2/PGE2 levels
EP1	Gq	Ca2+ ↑	Variable	COX-2 upregulates EP1 and EP1 upregulation corresponds with COX-2 downregulation (when AA ↓)
EP2	Gs	cAMP ↑	Frequently ↑	EP2 signaling often supports COX-2 upregulation
EP3	Gi (or Gs/Gq)	cAMP↓	Often ↓ or unchanged	EP3 typically suppresses cAMP and COX-2, chronic stimulation may lead to desensitization of EP3
		(Ca2+ ↑)		
EP4	Gs/or β-arrestin-coupled	cAMP↑	Frequently ↑	EP4 strongly promotes COX-2 expression and PGE2 synthesis
references	[21]	[21]	[34,35]	[28,34,35]

Thus, sustained COX-2-dependent PGE2 production favors Gs-coupled EP2 and EP4 signaling while constraining Gi-linked EP3. When cAMP upregulation is no longer restrained by EP3 sustained PGE2 production by cAMP could be expected.

For further details, see the review of prostanoid receptors by Biringer (2021) [21].

### 3.2 Terminology

Hereafter, we use EP3 as shorthand for “PGE2 activity through EP3”. We discuss how receptor activity could differ among individuals. We will often refer in this article to “EP receptor “activation capacity” or “capacity” instead of “EP receptor expression”, because their potential activity depends on many other factors. Given their effects on cAMP, we assume that enhanced or insufficient EP3 signaling broadly corresponds to reduced or increased EP2/EP4 signaling. We refer to these conditions as “EP3 up” or “EP3 down”, respectively.

## 4 The reported role of PGE2 in COVID-19

As noted above, PGE2 signaling through the EP3 receptor has been implicated in mediating the general symptoms of viral infections, including COVID-19. Excessive PGE2 activity has also been reported to contribute to tissue and organ injury. Aliabadi et al. described in 2020 multiple forms of COVID-19-associated organ damage, linked to PGE2 overactivity acting through distinct EP receptors [6]. Their work provides a comprehensive overview of established downstream mechanisms of PGE2-mediated pathology in acute COVID-19 infection. However, the factors that may account for the increased severity and apparently higher frequency of these manifestations in COVID-19, compared with other coronavirus infections, remained incompletely understood.

### 4.1 How might SARS-CoV-2 provoke more intense inflammatory reactions than other coronaviruses?

• The SARS-CoV-2 spike protein is reported to upregulate PGE2 [36]. Impaired immune response mediated by PGE2 promotes severe COVID-19 disease [5].
• The entry point of SARS-CoV-2 is the angiotensin-converting enzyme 2 (ACE2) receptor. Soluble ACE2 is dysfunctional in COVID-19 [37], resulting in upregulation of Ang II [38].
• Ang II has been reported to stimulate PGE2 production through its AT1 receptor [39].
• PGE2 stimulates renin, renin stimulates Ang II [40], and Ang II stimulates PGE2 [39].
• Accordingly, after initial PGE2 upregulation by infection, a self-enhancing PGE2 loop could be established.
• ACh levels and lipid mediator levels are reportedly associated with COVID-19 severity [30]. PGE2 interacts with neural networks in a feedback loop with ACh [41,42]. ACh has also been reported to stimulate PGE2 [43,44].
• PGE2 stimulates cytokine production, and vice versa [45,46]. This reciprocal interaction may trigger an inflammatory cascade - the so-called cytokine storm - also described as an “eicosanoid storm” by Dennis et al. (2015) [47]. Notably, cytokines also stimulate the entry of PGE2 into the brain [48].
• By binding to EP2 and EP4, PGE2 can upregulate COX-2 [27], establishing another self-enhancing loop since COX-2 is required for PGE2 synthesis (section 3).
• Baseline PGE2 levels are reportedly higher in individuals with preexisting medical conditions, older adults, men [5], and those with increased adipose tissue, as PGE2 is stimulated by adipose tissue under pathological conditions [49]. These findings could help explain why individuals from these groups may be more susceptible to severe COVID-19.

### 4.2 Long COVID following a mild infection?

If marked upregulation of PGE2 is presumed to contribute to the development of LC, how can it be explained that most LC patients experienced only mild acute COVID-19 infection [50]? As noted above, many symptoms of viral infection are mediated by PGE2 signaling through the EP3 receptor. We propose that high PGE2 levels combined with increased EP3 capacity (“EP3 up”) may account for severe acute disease, whereas reduced EP3 capacity (“EP3 down”) may underlie mild or even clinically silent infection [4]. Even in the presence of significantly elevated PGE2, insufficient EP3 capacity may attenuate symptom expression, rendering acute infection mild or undetectable [51]. PGE2 binding to EP3 acts centrally to elicit sympathetic activation [52,53] and at parasympathetic terminals reduces ACh release [41]. Thus, “EP3 up” or “EP3 down” combined with upregulated PGE2 could explain frequent autonomic dysfunction in LC, by inducing either excessive or insufficient sympathetic or parasympathetic activity [54]. We propose that patients with an “EP3 up” or an “EP3 down” constitution may be more vulnerable to LC.

### 4.3 PGE2 upregulation underlying long COVID following viral clearance

How might PGE2 remain elevated after SARS-CoV-2 clearance?

Several hormones that normally stimulate PGE2 production are themselves inhibited by PGE2, hinting at feedback loops that could prolong its activity. Arginine vasopressin (AVP) illustrates this well: COVID-19 disrupts AVP function, contributing to inflammation [55], and “thirst” is a common long COVID symptom [56]. Elevated PGE2 can suppress AVP [57], yet AVP simultaneously promotes PGE2 production across multiple tissues [58,59]. Such reciprocal regulation could theoretically favor sustained PGE2 elevation (see section 6. and Appendix A, Multimedia component 2). Subsequent review indicated that a broader range of mediators frequently implicated in long COVID have likewise been reported to stimulate PGE2 synthesis - including acetylcholine (ACh) [43,44], angiotensin II (Ang II) [39,60], dopamine (DA) [61,62], fibroblast growth factor 23 (FGF23) [63,64], interferon-γ (IFN-γ) [65,66], and serotonin (5-HT) [67,68] - while also being functionally antagonized by PGE2 (see section 5.1).

Under physiological conditions, such attenuation may serve adaptive functions, for example by conserving energy through inducing fatigue and reducing activity until stress factors are resolved. However, in pathological or chronic inflammatory settings, this regulatory mechanism may become dysregulated, contributing to maladaptive outcomes. We have integrated these findings into the following hypothesis.

### 4.4 “The PGE2 hypothesis for long COVID”

After being upregulated by COVID-19 infection, sufficiently high levels of PGE2, “the principal lipid mediator of inflammation”, may antagonize the activity of endocrine hormones and other substances that spontaneously stimulate PGE2 production. PGE2 could potentially reduce their influence through several mechanisms, including suppression of their release, interference within overlapping subcellular signaling pathways, receptor phosphorylation, and altered autoantibody production or class switching. Such mechanisms may have evolved to conserve homeostasis or preserve energy during physical or mental stress. When PGE2 levels rise high enough to chronically antagonize these agents, we describe this state as “PGE2 dominance.”

Multiple factors can drive elevated PGE2, including glucose level, advanced age, adiposity, comorbidities, toxins, dehydration, and mental stress. Establishment of PGE2 dominance appears to depend on an imbalanced ratio of EP3 and EP2/EP4 receptors (“EP3 up” or “EP3 down”), whose opposing functions can disrupt homeostasis. In this state, PGE2-stimulating hormones and other substances are persistently upregulated in attempts to restore balance, yet remain functionally inhibited by PGE2. This may create self-sustaining feedback loops that maintain PGE2 overproduction.

Ultimately, this process may culminate in resistance to multiple hormones - a state we term “hormonal multi-resistance”-, driving persistent upregulation of PGE2. Such chronic pathological levels of PGE2 could explain long COVID symptomatology, given PGE2's wide-ranging functions.

### 4.5 “Hormonal multi-resistance” as a context-dependent survival mechanism?

A highly complex mechanism as the proposed “hormonal multi-resistance”, is unlikely to represent a simple evolutionary accident. We have suggested that elevated PGE2 signaling antagonizes multiple hormonal pathways, potentially functioning as an energy-conserving response to physiological stress. From an evolutionary perspective, a heightened form of multiple hormonal resistance may have been advantageous in contexts such as hibernation, where resistance to leptin, AVP [69] and insulin supports hyperphagia, elevated glucose levels, and fat accumulation. Consistent with this view, hibernation is associated with transient leptin and insulin resistance [70] as well as a shift toward fatty acid metabolism [70], which liberates AA, providing substrate for COX-2-mediated PGE2 synthesis (see section 3). Sustained PGE2 signaling - potentially driven by altered EP receptor balance - may reduce hormonal responsiveness, suppress sympathetic activity, and promote sleep-like states. While adaptive and reversible in hibernating species, prolonged activation in humans may be maladaptive, contributing to persistent homeostatic dysregulation. Although this framework remains speculative, it is supported by the comprehensive review of molecular adaptations in mammalian hibernators by van Breukelen and Martin (2002) [70].

## 5 COVID-19, long COVID, PGE2, imbalanced EP3 capacity and hormonal resistance, are they interrelated?

Research suggests that COVID-19, LC, and resistance phenomena such as type 2 diabetes (T2D) and obesity are interrelated [71]. Both PGE2 and lack or excess of EP3 receptor expression have been reported to play crucial, yet incompletely understood, roles in insulin resistance or (T2D) and leptin resistance or obesity [[72], [73], [74], [75], [76], [77]]. We propose that the initial upregulation of PGE2 induced by SARS-CoV-2 infection may, in some individuals, persist beyond the acute phase, associated with altered EP3 receptor function. This imbalance may pre-exist and/or be further amplified by sustained PGE2 elevation during infection (see section 3.1, Table 1). Such alterations in EP3 signaling could contribute to homeostatic dysregulation and presumed resistance phenomena, as discussed in the following sections.

### 5.1 How could PGE2 antagonize other mediators, promoting resistance?

We previously noted that several PGE2 stimulating agents and are also antagonized by PGE2, including acetylcholine (ACh) [41], angiotensin II (Ang II) [78], AVP [57], serotonin (5-HT) [79], dopamine (DA) [80], fibroblast growth factor 23 (FGF23) [81], and interferon-gamma (IFN-γ) [82]. We identified several pathways by which PGE2 could antagonize the activity of other agents.

1. Stimulating functional autoantibodies (aabs) to the receptors of other agents and their class switching (through EP2/EP4)
2. Stimulating phosphorylation of their receptors, possibly attracting autoantibodies (through EP1)
3. Downregulation of their secretion (through EP3)
4. Indirect functional antagonism of GPCRs through shared subcellular pathways

#### 5.1.1 Stimulating autoantibodies and their class switching

Upregulation of autoantibodies (aabs) of the immunoglobulin G (IgG) class to GPCRs has been observed in both COVID-19 [83,84] and LC patients [85,86]. Animals experimentally infected with SARS-CoV-2 also generate functional aabs against GPCRs [87].

PGE2 has been shown to stimulate the production and class switching of autoantibodies from the immunoglobulin M (IgM) to IgG [88] or IgE types [[89], [90], [91]], through the cAMP pathway (EP2/EP4) [25]. Aabs can modulate the binding of true ligands. We suggest that PGE2 may hinder ligand binding in COVID-19 and LC through aab production [92,93].

#### 5.1.2 Stimulating phosphorylation processes

Another pathway involves stimulation of phosphorylation processes by PGE2 [[94], [95], [96]]. We suggest that PGE2 could interfere with receptor binding of specific compounds by promoting receptor or ligand phosphorylation, including via the EP1 PKC pathway [97]. Phosphorylation has been reported to increase the binding of aabs by up to 2- or 7-fold [98,99].

#### 5.1.3 Downregulation of the secretion of other agents

PGE2 reportedly inhibits the secretion of other hormones through receptor EP3, such as: ACh [100], insulin [101] and dopamine [102], noradrenaline [103] and serotonin [103].

#### 5.1.4 Indirect functional antagonism of GPCR-ligands through shared subcellular pathways

In section 3, it has been discussed that EP receptors belong to the class of the G-protein coupled receptors (GPCRs). Reportedly, PGE2 dominance could interfere with the signaling of other GPCR-binding hormones that use the same downstream signaling pathways [104]. GPCR signaling depends on a finite pool of Gi or Gs proteins and other downstream second messengers. The EP3 receptor is Gi-coupled, and EP2 and EP4 are Gscoupled [21]. Many other monoamine receptors are Gi-, Gs-, or Gq-coupled as well, see Table 2.

**Table 2:** G-protein coupled receptors and their overlapping second messenger pathways.

GPCR ligands	Gi-coupled cAMP↓	Gs-coupled cAMP↑	Gq-coupled Ca^2+^	references
PGE2	EP3	EP2, EP4	EP1	
Dopamine	D2, D3, D4	D1, D5		105
Histamine	H3, H4	H2	H1	106
Serotonin	5HT-1, 5HT-4, 5HT-5	5HT-4, 5HT-6, 5HT-7	5HT-2, 5HT-4	107
Melatonin	MT1, MT2	MT1		108
(Nor)Adrenaline	α2/β2, β3	β1, β2, β3	α1	109

Relatively strong or chronic activation of an EP receptor (PGE2 dominance) could reduce monoamine signaling efficacy or modulate it by sequestering G-proteins and depleting other second messengers or through other GPCR signaling machinery [110]. Even though multiple hormones may activate the same canonical pathway, distinct biological outcomes could emerge from shared downstream mechanisms. When PGE2, for example, suppresses cAMP-PKA through EP3, this does not have exactly the same effect as DA doing so through D2. In GPCR signaling, specificity arises from receptor localization and trafficking, ligand concentration and exposure time, differential coupling to G proteins and β-arrestins, selective engagement of scaffolding proteins, and the cell-type-specific complement of downstream effectors [110].

Of significance: This crosstalk mechanism is expected to work both ways: A relatively high level of GPCR-hormones could, in turn, antagonize PGE2 signaling (see sections 9.2, 4.4).

### 5.2 How could PGE2 antagonize insulin and leptin activity?

PGE2 levels are reportedly upregulated in DT2 and obesity [111,112]. High levels of PGE2 are also known to inhibit insulin production and secretion [101]. Insulin and leptin are not GPCR-coupled, but it has been shown that phosphorylation of insulin receptors, contributes to insulin resistance [113], a process that could hypothetically be stimulated by PGE2 (see section 5.1.2). PGE2 may also antagonize leptin in several ways. A recent paper suggested that chronic activation of mechanistic target of rapamycin (mTOR) pathways causes leptin resistance, which could be reversed by the mTOR inhibitor rapamycin [114]. Notably, PGE2 is reported to activate mTOR pathways [115], and rapamycin is known to suppress PGE2 production [116]. PGE2 repeatedly has been reported to stimulate phosphorylation of signal transducer and activator of transcription 3 (STAT3) [117] and to upregulate suppressor of cytokine signaling 3 (SOCS3). Both compounds contribute to multisystem autoimmune disease [118] and play major roles in insulin and leptin resistance [[119], [120], [121]]. In adolescents with overweight or obesity, IgG anti-leptin antibodies have been detected [122]. As argued in section 5.1.1, PGE2 dominance could antagonize leptin receptor binding by stimulating sufficiently high levels of such autoantibodies.

## 6 The hypothetical role of imbalanced EP3 capacity in hormonal resistance

PGE2 and imbalances in EP3 and EP4 receptor activity, enhanced or insufficient EP3 activity, are implicated in insulin and leptin resistance [[72], [73], [74], [75], [76], [77]] though the mechanisms remain incompletely understood.

PGE2-EP3 signaling suppresses lipolysis [123] glycogenolysis [124], and insulin production and secretion [98], while enhancing blood osmolality [125], brown adipose tissue formation [126], vasoconstriction [22], and diuresis [22]. Remarkably, both increased and decreased activity of these processes can yield similar outcomes, including hyperglycemia, adipose tissue expansion, and hypovolemia [127].

These imbalances could create self-reinforcing feedback loops: hypovolemia and elevated glucose stimulate arginine vasopressin (AVP), while hyperglycemia also promotes leptin secretion. Both AVP [58] and leptin [128] enhance PGE2 production, and PGE2 is additionally upregulated by high glucose [129] and hypovolemia [130]. There is substantial evidence that PGE2 can antagonize AVP [57], insulin and leptin [[72], [73], [74], [75], [76], [77]].

Collectively, these reciprocal interactions suggest that imbalanced PGE2-EP3 signaling may perpetuate insulin and leptin resistance, forming a network of metabolic and hormonal feedback loops that amplify dysregulation of glucose and fluid homeostasis.

### 6.1 Hypothetical “hormonal multi-resistance”

**Fig. 1:**
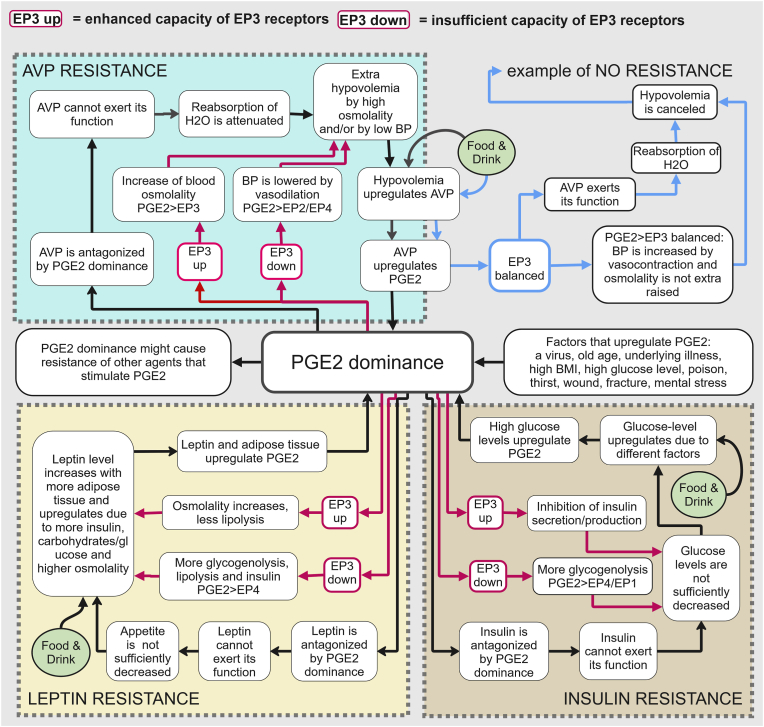
A hypothetical model of “hormonal multi-resistance”.

According to our hypothesis, PGE2 can antagonize the receptor binding of AVP, leptin, and insulin (while they presumably drive PGE2 upregulation as a limiter on their activity). These interdependent relationships may form vicious cycles - resistance loops - that potentially reinforce one another [131]. We refer to this presumed phenomenon as ‘hormonal multi-resistance”, illustrated in Fig. 1.

A combination of factors may cause such a strong upregulation of PGE2 that its levels begin to dominate over certain endocrine hormones that spontaneously stimulate PGE2 synthesis but can also be antagonized by it. In this state of “PGE2 dominance”, the functions of arginine vasopressin (AVP), insulin, and/or leptin may be suppressed. Given the functions of the PGE2 receptor EP3, homeostasis can become chronically disrupted in individuals with either “EP3 up” or “EP3 down” (enhanced or insufficient EP3 capacity), driving upregulation of PGE2. This, in turn, promotes resistance to hormones that are antagonized by PGE2 but stimulate PGE2, ultimately reinforcing resistance to additional hormones - a process we describe as “hormonal multi-resistance”. See for a detailed explanation including supporting references: Appendix A, Multimedia component 2: Hypothetical models of hormonal resistance to AVP, insulin, leptin, FGF23, IFN-gamma and serotonin.

**Fig. 2:**
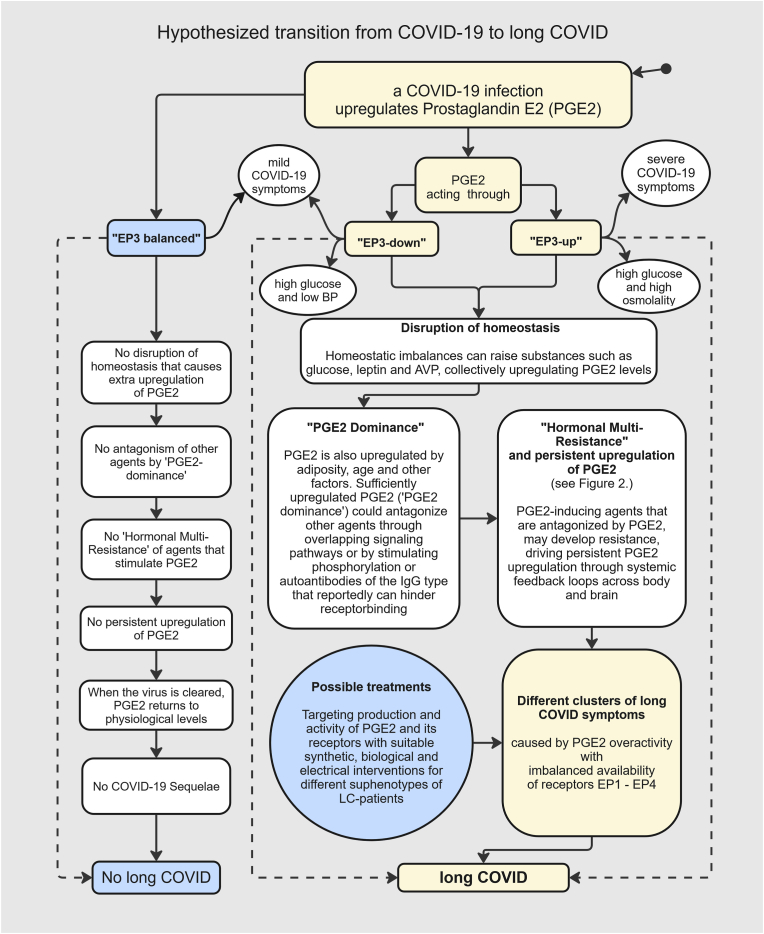
A graphical model of the PGE2 hypothesis for long COVID.

A hypothetical model of Long COVID driven by “hormonal multi-resistance”, is illustrated in Fig. 2. “A graphical model of the “PGE2 Hypothesis for long COVID”

Viral infections upregulate PGE2, including those caused by COVID-19. PGE2 is a multifunctional modulator of inflammation that binds to prostanoid receptors EP1, EP2, EP3, and EP4. Through receptor EP3 it causes, for example, general viral symptoms, raises osmolality and blood pressure, attenuates glycogenolysis, but decreases insulin production. In individuals with “EP3 up” or “EP3 down” (enhanced or insufficient EP3 capacity), homeostatic imbalances could lead to hormonal resistance that drive chronic upregulation of PGE2. This could offer a unifying explanation for the diverse symptomatology of long COVID.

## 7 Imbalanced EP receptor capacity and subphenotypes of patients

Such resistance loops, as discussed above, may remain “fueled” even after resolution of the initial stressors. However, persistent PGE2 dominance is expected only in individuals with an imbalance in EP receptor capacity, particularly involving EP3. This may explain why presumed sustained PGE2 upregulation and the subsequent development of long COVID occur in some individuals following COVID-19 infection but not in others.

### 7.1 Two phenotypes of obesity and diabetes Type 2: do they match hypothetical “EP3 up” and “EP3 down” receptor profiles?

Two opposing categories of T2D and obesity with shared biomarkers [132,133] have been identified:

Category A (compared to Category B): mostly male sex, lower insulin production, more insulin sensitivity, higher BP, lower glucose level, higher BMI, adipose tissue is more evenly distributed, less atherosclerosis.

Category B (compared to Category A): mostly female sex, more insulin production, less insulin sensitivity, lower BP, higher glucose level, adipose tissue is more ectopically distributed, and more atherosclerosis.

Other researchers have shown that T2D and obesity occur in groups of animals and humans with either enhanced [101] or insufficient [134] EP3 capacity.

We suggest that the characteristics of Category A can be explained by “EP3 up” and those of Category B by “EP3 down”, based on the functions that PGE2 stimulates through this receptor: see section 7.5, Table 3. Hypothetical biomarkers for two presumed categories of LC patients.

### 7.2 Correlation between long COVID symptom clusters and different EP receptor expressions?

Zhang et al. investigated the incidence and reproducibility of specific long COVID-symptoms in individuals who contracted COVID-19 (in the pre-Omicron period) [11]. In a development cohort of 20.881 and a validation cohort of 13.724 patients, they identified over 137 long COVID symptoms and conditions in four reproducible subphenotypes, named: Type 1 “Cardiac and renal”, Type 2 “Respiratory, sleep and anxiety”, Type 3 “Musculoskeletal and nervous”, Type 4 “Digestive and respiratory”. If PGE2 is upregulated systemically, its receptors are expected to have a major influence. We propose that each of these categories is associated with the dominant expression of a specific EP receptor, here supported by a selection of references: Type 1 – receptor EP3 [125,[135], [136], [137], [138]]; Type 2 – receptor EP1 [[139], [140], [141]]; Type 3 – receptor EP2 [[142], [143], [144], [145]]; Type 4 – receptor EP4 [[146], [147], [148]].

Danesh et al. (2023) defined two clusters of long COVID based on comparable clinical characteristics that again seem to match specific EP receptor expression, named: Cluster 1 “Neuropsychiatric” (more female) and Cluster 2 “Pulmonary” (more male, “more often hospitalized”) [149]. We suggest that:

• Cluster 1 “Neuropsychiatric” matches Zhang's Types 2, Type 3 and Type 4 - correlating with “EP3 down” plus “EP1 up”, “EP4 up” and “EP2 up” respectively.
• Cluster 2 “Pulmonary” which is characterized by “more often hospitalized”, “cardiac and renal” and “pulmonary” problems, matches Zhang's Type 1, “cardiac and renal” (also more often hospitalized [11]), that is associated with “pulmonary” problems as well [11] - correlating with “EP3 up” (pneumonia, cough [150,151]). Further references for the hypothetical subphenotype linked EP expressions are presented in Appendix A, Multimedia component 3, section 1. “Hypothetical EP receptor distribution in four subphenotypes of LC patients”.

### 7.3 Two long COVID cytokine clusters, do they match EP receptor expression?

Kervevan (2023) measured two different cytokine clusters in LC patients not hospitalized for COVID-19: a so-called “Seronegative long COVID” type and a “Seropositive long COVID” type [152]. The first group (96% female) was named seronegative because it had significantly fewer antibodies to the SARS virus and lower levels of inflammation than the seropositive group (79% female). Cytokine patterns will differ when stimulated mainly through EP3 (mainly inflammatory) or through EP4 (mainly anti-inflammatory). In this study, it was emphasized that the seronegative type showed significantly lower CD4^+^ activity. PGE2 dose-dependently suppresses antigen-specific CD4^+^ T-cell responses through EP2/EP4 [153]. Here, both physiological and biochemical characteristics of the two types seemed to match an “EP3 down” and “EP3 up” profile, respectively (see overview in Table 3. In section 7.5).

### 7.4 Could gender and sex steroids contribute to differing EP3 capacity?

Male and female sexes appear to be overrepresented in the “EP3 up” and “EP3 down” categories, respectively. Greater EP3 expression in males and reduced EP3 expression in females may provide a mechanistic explanation for the following findings: Male patients are, on average, more vulnerable to severe COVID-19 than female patients [5] and among male infants, the mortality rate following infections is higher than among female infants [154]. The general symptoms of viral infection are stimulated through EP3 [4]. Among female LC patients and among female infants, the prevalence of autoimmune conditions is higher [154]. Autoimmune responses are primarily stimulated through EP4 and EP2 [25,89] (see section 5.1.1). Estrogen alone or in combination with progesterone has been reported to modulate EP receptor expression and mRNA levels in bovine tissues [155]. These findings raise the possibility that developmental or hormonal changes in sex steroid levels may also influence EP receptor balance in humans particularly during puberty. Within the framework of the PGE2 hypothesis, such sex-hormone-dependent modulation could contribute to sex-related differences in clinical presentation (see sections 7.1-7.3) and may also be relevant to age-specific patterns observed between younger children (6 **-** 11 years) and peri-pubertal adolescents (12 **-** 17 years) with Long COVID [156,157].

If women are indeed found to have, on average, significantly lower EP3 capacity compared to men, leading to proportionally greater capacity through EP2 and EP4, this could help explain sex-based medical differences observed in LC and its comorbidities. For more detailed information on hypothetical EP receptor expression clusters and supporting references, see Table 3 and Appendix A, Multimedia component 3, section 1.

### 7.5 Hypothetical biomarkers for two presumed categories of LC patients

We listed aforementionedbiomedical characteristics that match PGE2 activity through "EP3 up" or “EP3 down” in Table 3.

**Table 3:** Hypothetical biomarkers for two presumed categories of long COVID patients, “EP3 up” and “EP3 down”

Hypothetical biomarkers for long COVID Category A	Hypothetical biomarkers for long COVID Category B	references
“EP3 up” enhanced capacity of EP3∗	“EP3 down” insufficient capacity of EP3∗	
∗ EP3-receptor capacity as compared to healthy subjects		
data compared to presumed category B patients:	data compared to presumed category A patients:	
higher percentage was hospitalized for COVID-19	higher percentage experienced a mild COVID-19 infection	[11]
higher percentage of male patients	higher percentage of female patients	[11,149]
more antibodies against Sars	less antibodies against Sars	[152,238,239]
more activity of CD4^+^ cells	less activity of CD4^+^ cells [161]	[153]
more proinflammatory activity	more anti-inflammatory activity	[153]
more sympathetic activity	less sympathetic activity	[52]
can develop a high fever	not prone to high fever	[51,240]
higher blood pressure - measured in adolescence (before age-induced changing PGE2 levels that could stimulate BP and atherosclerosis)	lower blood pressure - measured in adolescence (before age-induced changing PGE2 levels that could stimulate BP and atherosclerosis)	[22,241]
more thrombotic problems	less thrombotic problems	[164]
less lipolysis, more evenly distributed (brown) adipose tissue [126]	more lipolysis leading to more ectopic adipose tissue [165]	[166]
more insulin sensitivity	less insulin sensitivity	[165]
attenuated insulin secretion	insulin secretion is less attenuated	[167]
less β-cell proliferation and survival	more β-cell proliferation and survival	[168]
lower glucose level	higher glucose level	[169]

## 8 Hypothesis testing

### 8.1 Assessing prostaglandin E-major urinary metabolite concentrations

Establishing elevated, stable prostaglandin E2 Major urinary metabolite (PGE2-M/PGE2-MUM) levels could be an initial step. Groups of LC patients could be compared with age- and sex-matched control groups, as basal PGE2 levels differ between the sexes. PGE2 levels are lower in young females compared to young males, increase in females, and decline in males with aging [158].

### 8.2 Comparing EP receptor densities

We have noted that high levels of PGE2 can downregulate EP3 expression and upregulate EP1, EP2, and EP4 expression (see section 3.1, Table 1). Within-patient samples could be compared, biopsies [158] taken before before infection and after LC diagnosis to evaluate changes in EP receptor expression. Our hypothesis is that two categories will emerge: one, “EP3 up” (Category A), will have significantly higher EP3 expression than the other, “EP3 down” (Category B), before and after infection. To verify the presence of “EP3 up” or “EP3 down” profiles, and possibly other unbalanced EP expression profiles in LC, the receptor densities of EP1-EP4 could be assessed in age- and sex-matched patient and healthy control groups. Matching is important, since EP3 receptor density decreases with aging has been reported [158], and our hypothesis presumes sex-based differences in EP receptor expression. Important to note: if the regulatory effects of chronic PGE2 exposure on EP receptor expression are applicable in this context, it is conceivable that most individuals, in Category A as well, would be expected to display reduced EP3 expression, with EP1 upregulation potentially present across both cohorts.

#### 8.2.1 Comparing mitochondrial EP3 densities

Mitochondrial EP3 density in long COVID (LC) patients is also presumed to be lower compared to controls and pre-infection biopsies. We base this assumption on the following data: Upregulated PGE2 has been reported to impair mitochondrial function [159,160]. PGE2 signaling through EP2 and EP4 has been reported to play a role in disrupting mitochondrial function: Morotti et al. identified the PGE2 - EP2/EP4 signaling axis as a key mechanism underlying mitochondrial depolarization [161] and, as mentioned, high levels of PGE2 have been shown to downregulate EP3 expression on plasma membranes [161] Downregulation of EP3 could be a mechanism by which upregulated PGE2 causes mitochondrial dysfunction and, as a result, chronic fatigue - the signature symptom of long COVID.

#### 8.2.2 Density of all four EP receptors should match specific symptom clusters

Our hypothesis suggests that the expression pattern of all four EP receptors (EP1, EP2, EP3, and EP4) in LC patients should match specific symptom clusters experienced by these patients. According to published data, these symptom clusters could be linked to specific distributions of these receptors. This could be investigated. For further details, see Appendix A, Multimedia component 3, section 1.

## 9 Clinical implications of a validated PGE2 hypothesis

Our hypothesis, if validated, could offer many diverse options for different subphenotypes of long COVID patients. If imbalanced EP receptor activation potential, combined with sustained upregulation of PGE2, is shown to drive “hormonal multi-resistance” underlying long COVID symptoms and comorbidities, then therapeutic strategies should focus on reducing PGE2 levels and rebalancing receptor activation capacity. Further details are available in Appendix A, Multimedia component 3, section 2.

### 9.1 Side effects of NSAIDs or corticosteroids and EP receptor imbalance?

SAIDs and corticosteroids are known to inhibit COX-2 and PGE2 production. However, these drugs are not always beneficial. Two non-exclusive explanations may account for these observations. First, NSAIDs reduce not only PGE2 but also other COX-2-derived prostaglandins with important physiological roles, including PGI2 (vasodilatation, anticoagulation) and TXA2 (vasoconstriction, coagulation) [162]. Second, lowering PGE2 levels does not necessarily correct imbalances in EP receptor expression and may even exacerbate disparities in EP3 versus EP2/EP4 signaling: COX-2/PGE2 downregulation is expected to favor increased EP3 expression with concomitant reductions in EP1, EP2, and EP4 (see section 3.1). Consequently, NSAID-associated adverse effects may reflect altered contributions of multiple COX-2-derived ligands combined with shifts in EP receptor expression.

Consistent with this notion, well-known NSAID side effects are mediated by PGE2 through EP3, such as cardiovascular and renal problems [125,[135], [136], [137], [138],^163^] or gastrointestinal issues linked to impaired mucosal protective signaling, potentially due to reduced EP4 capacity [164]. Individuals with “EP3 up” may be more prone to NSAID side effects, while other EP receptor imbalances can also trigger adverse effects after COX-2/PGE2 inhibition, due to overactive or insufficient activity of specific prostaglandins. Thus, it might be safer to use COX-2 inhibitors in combination with specific EP receptor antagonists and agonists to decrease averse PGE2 activity. Such options are being researched [162]: see Appendix A, Multimedia component 3, section 2.

For more information on potential EP receptor modulation, see Appendix A, Multimedia component 3, section 2.

### 9.2 The potential benefit of monoamines to reduce PGE2 signaling

Both PGE2 and monoamines act as GPCR ligands, sharing downstream signaling pathways (Gi, Gs, Gq) as described in section 5.1. Upregulation of PGE2 may therefore suppress monoaminergic signaling, while monoamines could reciprocally attenuate PGE2 activity. Melatonin, via its MT1 receptor, is known to antagonize PGE2 activity [165], suggesting that dopamine, serotonin, and noradrenaline might exert similar modulatory effects. This competition for shared G-protein signaling could underlie the anti-inflammatory and analgesic actions of these monoamines. Accordingly, targeted pharmacological enhancement of specific monoamines may help counteract excessive PGE2-mediated inflammation. For these and other proposed clinical consequences of a validated hypothesis, see Appendix A, Multimedia component 3, section 2.

## 10 PGE2 and dopamine: are they modulators of each other's activity?

PGE2 and DA reportedly interact with each other's receptors and production.

• PGE2 is a major mediator of inflammation [25].
• DA has anti-inflammatory effects [166].
• Through its receptor EP3, PGE2 mediates systemic sickness responses to respiratory virus infection [4]. Research shows that malaise and aversion could be caused in humans by upregulated levels of PGE2 because it attenuates DA activity [167].
• Through the EP3 receptor, DA release can be inhibited by PGE2 [102].
• Receptor EP1 renders dopaminergic neurons selectively vulnerable to direct PGE2 neurotoxicity [168].
• Through receptor EP1, DA activity can be downregulated by PGE2 in other ways [80,141,169].
• Lack of EP1 can lead to a hyperdopaminergic state in mice [170].
• Through EP1, COX-2/PGE2 can be downregulated by an unknown ligand [28,171].
• GLP-1 can reduce arachidonic acid (AA), COX-2, and PGE2 [172,173]. Researchers propose that it is through this PGE2-reducing mechanism that GLP-1 increases dopamine signaling in the brain [174].

### 10.1 Cognitive dysfunction and brain fog, possibly explained by PGE2-DA interaction?

One of the frequent symptoms of LC is brain fog [175]. PGE2 decreases DA activity in several ways and in diverse tissues, including in the brain, through receptor EP1. COX-2 upregulates EP1 [21]. Thus, EP1 is expected to be upregulated when PGE2 and COX-2 remain chronically elevated. Consequently, persistently high levels of PGE2 may reduce DA activity, which is critical for cognitive performance [176]. Supporting this interpretation, meloxicam, a selective COX-2 inhibitor, has been reported to significantly protect diabetic rats from cognitive impairment [177].

This link between PGE2, DA, and cognition also provides insight into the frequent co-occurrence of brain fog and chronic pain [178], since PGE2 is the most influential lipid mediator contributing to inflammatory pain [179].

### 10.2 Olfactory dysfunction, PEM, and POTS: Potential role of PGE2 - DA interactions?

Extending this line of reasoning, we propose that PGE2 - DA interaction, in the context of imbalanced EP receptor expression, could also account for additional LC manifestations and comorbidities that remain inadequately understood. These include loss of smell and taste [180,181], postural orthostatic tachycardia syndrome [182,183], and post-exertional malaise (PEM) [184]. Together, these observations strongly suggest that PGE2-DA interactions, modulated by EP receptor balance, represent a mechanism that could underlie several hallmark symptoms of long COVID. For further clarification and supporting references: see Appendix A, Multimedia component 4: PGE2 - DA interactions, relevant for olfactory dysfunction, PEM, and POTS?

### 10.3 (Partially) successful long COVID interventions; PGE2 hypothesis-driven mechanistic considerations

In Table 4, we summarize several therapeutic approaches for long COVID that have demonstrated at least partial efficacy in patients. Our hypothesis may help explain this partial efficacy. First, we propose that PGE2 plays a principal role in LC and that EP receptor expression differs among patients, which may necessitate different therapeutic interventions. Second, as mentioned in the previous paragraph regarding COX-2-lowering medications, a substantial decrease in PGE2 levels could upregulate EP3 receptors while downregulating EP1, EP2, and EP4 (see section 3.1). Imbalances in EP expression could be reduced (especially in Category B - “EP3 down”) but also exacerbated (especially in Category A - “EP3 up”) (see Table 3 in section 7.5). Consequently, certain PGE2-lowering interventions may be beneficial for some patient subsets but ineffective - or even harmful - for others.

**Table 4:** (Partially) successful long COVID interventions; PGE2 hypothesis-driven mechanistic explanations.

Long COVID therapy trials	Published data	Hypothetical molecular mechanisms underlying symptom alleviation in LC
**DNA Aptamer drug BC007** **Rovunaptabin** Two cohort studies [185,186] and a case report [187] “The removal of GPCR-AAb might ameliorate the characteristics of the LCD, such as capillary impairment, loss of taste, and CFS.” [187]	BC007 decreases IgG-autoantibody-activity [187] PGE2 stimulates autoantibodies through EP2 and EP4 [[88], [89], [90], [91]]	BC007 could decrease inflammatory PGE2 activity, neutralizing autoantibodies (see section 5.1.1) potentially beneficial for patients with “EP2 up” and/or “EP4 up” (“EP3 down”)
**Histamine receptor antagonists (HRA)** Cohort study [188] “72% of patients with long COVID who received HRA reported clinical improvement.”	HRA could decrease inflammatory PGE2 activity [188] Histamine reportedly upregulates COX-2/PGE2 through H1[189] and through H2 [190] HRA therapy antagonizes histamine activity [188]	HRA could decrease inflammatory PGE2 activity by preventing histamine from upregulating PGE2 through H1 and H2 potentially beneficial for patients with “EP3 down”
**Hyperbaric oxygen (HBOT)** Cohort study [191] Review [192] “56-63% of long term-ill patients had a clinically relevant improvement in MCS/PCS 3 months after HBOT. **However, 13**–**19% of the patients had a clinically relevant deterioration in MCS/PCS**.” [191]	HBOT decreases PGE2 level [[193], [194], [195]] HBOT alleviates predominantly cognitive symptoms [191] that we presume to be stimulated by EP1 (see section 10. And 11.1) “The symptoms that showed most improvement were predominantly in the cognitive domain [191].”	HBOT decreases inflammatory PGE2 activity effectively [[193], [194], [195]] HBOT elevates BP [196] BP is elevated through EP3 [22] potentially beneficial for patients with “EP1 up” potentially **harmful for patients with “EP3 up” & “EP1 down”** potentially not harmful for patients with “EP3 down”
**Lidocaine** Subcutaneous lidocaine-HP-β-CD: every other day or daily Cohort study [197] “Twenty-seven of 30 symptoms improved significantly at week 24 of treatment compared to pre-treatment. No serious adverse events were reported.” [197]	Prolonged Lidocaïne administration reportedly decreases COX-2/PGE2 activity [198]	Prolonged Lidocaïne administration could hypothetically alleviate long COVID symptoms decreasing PGE2 activity by binding to the Gq subunit of EP1 (see section 11.1) potentially beneficial for patients with “EP1 up” potentially not beneficial for patients with “EP1 down”
**Low Dose Naltrexon** Cohort study [199] Case series with CFS patients [200] “We found a subset of 52 % of patients to be responders after 12 weeks of treatment.” Case series with CFS patients [200] “small-scale clinical trials have shown efficacy and low toxicity.”	LDN decreases PGE2 activity through antagonizing TRL-4 activity [201] TRL-4 triggers PGE2 production [116]	LDN could decrease inflammatory PGE2 activity through antagonizing TRL-4 activity [201] potentially beneficial for patients with “EP3 down”
**Stellate Ganglion Block (SGB)** Cohort study [202] “Results indicated that a substantial proportion of patients (86%) experienced a reduction of their symptoms following SGB treatment.” [202]	In general, the SNS and PNS have opposing effects, and they interact [203] possibly through PGE2 activity: PGE2 binding to EP3 increases inflammatory SNS activity 52 53 and suppresses PNS activity [41] (see section 4.)	SGB could decrease inflammatory PGE2 activity by inhibiting inflammatory SNS signaling [204] and stimulating anti-inflammatory PNS signaling may provide benefits across the four subphenotypes
**Vagal Nerve Stimulation (VNS)** Cohort study [205] “This pilot study provides preliminary evidence supporting the potential of t-VNS as a therapeutic intervention for female Long COVID patients.”	VNS decreases brain COX-2 expression [206] PGE2 binding to EP3 suppresses PNS activity [41] and increases inflammatory SNS activity 52 53 (see section 4.)	VNS could decrease inflammatory PGE2 activity by stimulating anti-inflammatory PNS signaling decreasing inflammatory SNS activity may provide benefits across the four subphenotypes

We propose that interventions which reduce PGE2 levels while markedly increasing blood pressure may be more beneficial for the “EP3 down” category, given that PGE2 stimulates BP primarily via EP3 signaling [22]. In this context, a reduction in PGE2 activity would not be expected to increase BP unless EP3 capacity is significantly enhanced. Conversely, interventions that attenuate PGE2 signaling within the sympathetic nervous system (SNS) may be more appropriate for the “EP3 up” category, where they could help modulate excessive EP3-mediated signaling. For hypothetical EP3 up or EP3 down biomarkers, see Table 3 in section 7.5.

For clinical consequences of a validated hypothesis, see Appendix A, Multimedia component 3, section 2.

### 10.4 Could lidocaine function as an EP1 antagonist?

Available evidence suggests that lidocaine can bind to the Gq subunit of EP1, thereby inhibiting downstream increases in Ca2+ levels, P2X7 activity, COX-2/PGE2 levels, and EP1-mediated PGE2 signaling. We propose a central role for EP1 in brain fog (see Section 10.1). Clinically, lidocaine has been reported to reduce concentration problems [197]. Non-responders to chronic lidocaine treatment may have insufficient EP1 receptor expression. For a detailed, extensively referenced discussion, see Appendix A, Multimedia component 3, section 2.2: Could lidocaine function as an EP1 antagonist, decreasing Ca2+ levels?

## 11 Discussion

Numerous mechanisms have been proposed to contribute to the development of long COVID. An open question is whether these diverse processes might be linked, at least in part, through PGE2-related signaling. Currently, post-acute inflammation, immune dysregulation, viral persistence, latent virus reactivation, thrombosis, dysbiosis, and mitochondrial dysfunction are among the leading pathophysiological mechanisms proposed to underlie the broad spectrum of long COVID symptoms. We examined these biological mechanisms as summarized in a recent review on the pathophysiology of long COVID by Peluso and Deeks in *Cell* [1]. While these mechanisms are often discussed independently, available literature suggests that they all may be compatible with, and potentially influenced by, PGE2-mediated processes. Thus, these established models and other molecular explanations proposed for long COVID symptoms can be considered within the context of our PGE2-centered hypothesis (see Table 5 below) “Documented links between proposed mechanisms for long COVID and PGE2 activity”.

**Table 5:** “Documented links between proposed mechanisms for long COVID and PGE2 activity”.

The biological mechanisms of long COVID according to Peluso's Figure 4 [1].	Separate articles on the proposed mechanisms for the pathophysiology of long COVID	Documented links with PGE2 activity
**Virus persistence**	“Long COVID: A proposed hypothesis-driven model of viral persistence for the pathophysiology of the syndrome” [207]	PGE2 has been shown to stimulate virus persistence [208]
**Post-acute inflammation**	“Long-COVID and Post-COVID Health Complications: An Up-to-Date Review on Clinical Conditions and Their Possible Molecular Mechanisms” [209]	PGE2/COX-2 is reported to stimulate proinflammatory cytokines that stimulate PGE2/COX-2 46,[210]. Such positive feedback loops could keep proinflammatory cytokines upregulated in LC
**Autoimmunity**	“Autoimmunity in long COVID” [85]	PGE2 is reported to induce autoimmune diseases [25,211] and to stimulate IgM autoantibodies and class switching to IgG autoantibodies [90,212] Inhibition of cyclooxygenase-2 blunts human B-cell antibody production [213]
**Thrombosis**	“COVID-19 and Long-COVID Thrombosis: From Clinical and Basic Science to Therapeutics” [214]	PGE2 stimulates coagulation through EP3 164 and stimulates COX-2 through an EP2/EP4-pathway [27]. Thus, as TXA2 is a COX-2 product [214], PGE2 is expected to stimulate TXA2 production
**Latent virus activation**	“Lights and Shadows of Long COVID: Are Latent Infections the Real Hidden Enemy?” [215]	PGE2 has been shown to activate latent viruses [208]
**Dysbiosis and gut translocation**	“The Microbiota in Long COVID” [216]	PGE2 through its receptor EP4 diminishes T(reg)-favorable commensal microbiota and plays a major role in IBD [[217], [218], [219]] and gut translocation [220].
**Mitochondrial dysfunction**	“Mitochondrial dysfunction in long COVID: mechanisms, consequences, and potential therapeutic approaches” [221]	PGE2 has been shown to cause mitochondrial dysfunction [159,222,223]

**Other proposed biological mechanisms for long COVID**	Articles on the proposed mechanisms for the pathophysiology of long COVID	Connections to the PGE2 -hypothesis for long COVID

**Endothelial dysfunction**	“The Significance of Endothelial Dysfunction in Long COVID-19 for the Possible Future Pandemic of Chronic Kidney Disease and Cardiovascular Disease” [224]	PGE2 is reported to cause endothelial dysfunction [225,226]
**Upregulation of Calcitonin gene-related peptide (CRGP)**	“Calcitonin gene-related peptide-induced central sensitization: A hypothesis for long COVID symptoms” [227]	PGE2 is reported to upregulate Calcitonin gene-related peptide (CGRP) [[228], [229], [230]]
**Serotonin reduction**	“Serotonin reduction in post-acute sequelae of viral infection” [231]	PGE2 antagonizes release and production of serotonin [79,232]
**Autonomic dysfunction**	“Cardiovascular autonomic dysfunction in post-COVID-19 syndrome: a major healthcare burden” [233]	PGE2 modulates the autonomic nervous system and cardiovascular regulation through EP3 [51,234,235].
		When EP3 expression is imbalanced and PGE2 is upregulated, this could lead to cardiovascular autonomic dysfunction

The PGE2 hypothesis for Long COVID is integrative but has several limitations. While studies report elevated PGE2 and related prostanoids in acute COVID-19 and Long COVID [7,8], systematic longitudinal profiling across disease stages is lacking, leaving temporal dynamics unresolved. Much supporting evidence comes from non-SARS-CoV-2 contexts, so direct causal links in Long COVID remain unproven; the hypothesis should thus be seen as a unifying, testable framework. PGE2 is part of a complex inflammatory network, and its dysregulation may reflect upstream changes in cyclooxygenase activity, arachidonic acid metabolism, immune cell composition, or other eicosanoids [10,236]. Finally, persistent PGE2 upregulation and receptor imbalances are hypothetical and require validation through patient stratification, receptor analyses, functional assays, and longitudinal studies to determine clinical relevance.

If proven valid, the PGE2 theory for long COVID symptomatology highlights an essential missing part of the pathophysiology of long COVID and its comorbidities. Our hypothesis offers explanations for the various LC symptoms and the different sets of LC symptoms reported. When researchers take on the challenge of measuring EP receptor density across different patient groups to confirm predictions, this could have substantial implications for finding treatments for LC and other chronic fatigue syndromes. Approaches to target PGE2 synthesis and signaling, as well as EP receptors with appropriate agonists and antagonists, could be developed [237]. We also hope to see the identification of biomarkers to help select potentially suitable treatments for different patient categories.

## Ethics statement

Ethical approval was not required because no clinical research involving human participants or materials was conducted. This work solely involves theoretical analysis of published research.

## No ethics statement needed

This manuscript does not need an ethics statement. No patients, patient material or new animal research were involved. Only published data have been assembled and analized.

## Funding

This research did not receive any specific grant from funding agencies in the public, commercial, or not-for-profit sectors.

## CRediT authorship contribution statement

**Vjera Ramer:** Conceptualization, Investigation, Visualization, Writing – original draft, Writing – review & editing. **Gert A. van Montfrans:** Supervision, Writing – original draft, Writing – review & editing.

## Declaration of competing interest

The authors declare that they have no known competing financial interests or personal relationships that could have appeared to influence the work reported in this paper.

## Data Availability

No data was used for the research described in the article.
