# Supplementary material for: Persistent prostaglandin E2 upregulation and hormonal multi-resistance: A hypothesis for long COVID
Source: Biochem Biophys Rep. 2026 Mar 10;46:102518. doi: 10.1016/j.bbrep.2026.102518 (PMC12994086; doi:10.1016/j.bbrep.2026.102518)
Supplement: Multimedia component 1 [file mmc1.docx]

Multimedia component 1. The Wideranging Functions of Prostaglandin E2

| **Section 1. Long COVID symptoms and comorbidities stimulated by PGE2** | | | |
| --- | --- | --- | --- |
| Apnea^(1)^  Anemia^(2)^  Atherosclerosis^(3)^  Asthma^(4)^ Alzheimer^(5)^  Autonomic Dysfunction^(6)^ Bronchoconstriction^(7)^ Cancer^(8)^  Cough^(9)^  Cardiac Hpertrophy^(10)^  Cardiomyopathy^(11)^ Type 1 Diabetes ^(12)^  Type 2 Diabetes ^(13)^  Dysmenorrhoea^(14)^  Depression^(15)^  Endothelial Dysfunction^(16)^  Gastro-intestinal inflammation ^(17)^  IBS^(18)^ Low Blood Pressure^(19)^  Lymphopenia^(20)^  Malaise/Aversion^(21)^  Migraine^(22)^  Mitochondrial Dysfunction^(23-25)^  Muscle weakness^(26)^   Myalgia^(27)^  Obesity^(28)^  Nausea^(29)^  Neurological conditions^(30)^  Osteoarthritis^(31)^  Pain^(32)^  Parkinson^(33)^  (Acute) Kidney Injury^(34, 35)^  Respiratory problems (Asthma)^(36) (37)^  Sleep/Wake problems via EP2/COX2/PGD2^(38)^  Thirst^(39)^  Thrombotic problems via EP3^(40)^  and via EP2>COX2>TXA2^(49)^  *There are more studies available that link these and other LC symptoms to PGE2 activity* | | | |
| **Section 2. Prostanoid receptors EP1 -EP4 ^(41-43)^, an overview** | | | |
|  | | | |
| **EP3**^(41-43)^ | **EP4**^(41-43)^ | **EP2**^(41-43)^ | **EP1**^(41-43)^ |
| EP3 has the most affinity for PGE2 of these four receptors | EP4 has less affinity for PGE2 than EP3 | EP2 has less affinity for PGE2 than EP3 | PGE2 binds the weakest to EP1 |
| G-proteïn-coupled or CA2+ stimulated | G-proteïn-coupled | G-proteïn-coupled | CA2+ stimulated |
| cAMP downregulation | cAMP upregulation | cAMP upregulation | cAMP downregulation |
| Eight splice variants of this receptor have been identified, some *upregulate* cAMP^(44, 45)^ | EP4 desensitizes quickly (when stimulated by a sufficient high level of PGE2 it rapidly internalizes: it moves to the inside of the cell membrane) | EP2-function is often similar to EP4- function, PGE2-action through EP2 lasts longer | Two splice variants have been identified. EP1- function is often similar to EP3- function |
| Contraction | Relaxation | Relaxation | Contraction |
| Contraction of skeletal muscles | Relaxation of skeletal muscles | Relaxation of skeletal muscles | Contraction of skeletal muscles |
| Contraction of smooth muscle | Relaxation of smooth muscle | Relaxation of smooth muscle | Contraction of smooth muscle |
| Vasoconstriction | Vasodilatation | Vasodilatation | Vasoconstriction |
| Increasing BP | Decreasing BP | Decreasing BP | Increasing BP |
| Diuretic^(46)^  Antidiuretic under high salt conditions | Antidiuretic^(46)^  Increase renin release | Antidiuretic^(46)^  Increase renal blood flow | Diuretic^(46)^  Stimulating renin |
| PGE2 the major lipid mediator of inflammation stimulates | | | |
| through EP3 | through EP4 | through EP2 | through EP1 |
| inflammatory processes, ao upregulating inflammatory cytokines such as il-6 ^(47)^ | anti-inflammatory processes, ao downregulating CD4+ activity, stimulating anti-inflammatory cytokines such as il-10^(48)^ | by stimulating COX > upregulation of its own production and that of other prostaglandins of the 2-series like PGD2, PGI2 and TXA2^(49)^ | an unknown ligand through EP1 can attenuate PGE2 activity through degradation of COX-2^(52)(53)^ |
| induces acute and chronic inflammation, through mast cell activation and T helper differentiation respectively ^(50)^  regulates expression of COX-2 (cyclooxygenase-2) in the kidney ^(51)^ | induces chronic inflammation and various autoimmune diseases through EP4 and EP2 receptors^(50)^ |  |  |
| All four EP-receptors are expressed in the brain and the CNS and in all cells on the membranes of mitochondria ^(54)^ and on the nuclear envelope ^(55)^ | | | |
| Expression of EP3  In the brain and the CNS and on the membrane of cells in the majority of tissues | Expression of EP4  In the brain and the CNS and on the membrane of cells in the majority of tissues | Expression of EP2  In the brain and the CNS plus EP2 expression is detected in lung, kidney, intestinal, glandular tissues, bone marrow, spleen, thymus, tonsils, mucous membranes, and skin | Expression of EP1  The EP1 receptor mRNA is ubiquitously expressed and EP1 receptor functions as a constrictor in the smooth muscles of the trachea, gastrointestinal tract, bladder, uterus and is especially expressed in the nervous system. |
| all four EP-receptors stimulate cell-proliferation in specific types of cancer | | | |
| PGE2 through EP3 | PGE2 through EP4 | PGE2 through EP2 | PGE2 through EP1 |
| *Metabolic*  ▪ Apoptosis of insulin- producing bèta-cells^(12)^  ▪ Negatively regulates glucose-stimulated insulin secretion (GSIS)^(56)^  ▪ Attenuates lipolysis^(57-59)^  ▪ Attenuates glycogenolysis ^(60)^ | *Metabolic*  ▪ Protects against apoptosis of insulin- producing bèta-cells^(61)^  ▪ Regulates bile acid synthesis, protecting against hypercholesterolemia^(62)^ |  |  |
| *Apoptotic*  ▪ Apoptosis of insulin producing bèta-cells^(12)^  ▪ Apoptosis of neutrophils^(63)^ | *Apoptotic*  ▪ Protects against apoptosis of insulin producing bèta-cells^(61)^ | *Apoptotic*  ▪ Stimulates apoptosis of oligodendrocyte precursor cells (OPC)^(64)^ that produce myelin  ▪ Apoptosis of neuronal cells in skeletal-muscle^(65)^ | *Apoptotic*  ▪ Apoptosis of dopaminergic cells in the substantia nigra^(66, 67)^ |
| *Brain processes*  ▪ Inhibition of noradrenergic neurons in the LC^(68)^ | *Brain processes*  ▪ Vasodilation involved in migraine^(22)^  ▪ Anxiolytic-like activity^(69)^ | *Brain processes*  ▪ Demyelination of nerves, neurotoxicity / cognitive decline by impairing myeloid metabolism^(70-73)^  ▪ Regulates neurotoxicity induced by aggregated alpha-synuclein^(72)^ | *Brain processes*  ▪ GABA -mediated inhibition of dopaminergic neurons in the midbrain^(69)^  ▪ inflammation-induced aversion^(74,75)^  ▪ Anxiolytic-like activity^(69)^  ▪ Suppressing impulse behaviour^(76)^ |
| *Nervous system*  ▪ Inhibiting endogenous pain control ^(77)^  ▪ Modulating signaling in the autonomic nervous system (PSN and SNS)^(6, 78)^ | *Nervous system*  ▪ Inflammatory pain^(79)^ | *Nervous system*  ▪ Stimulates apoptosis of oligodendrocyte precursor cells (OPC)^(64)^ that produce myelin  ▪ Apoptosis of neuronal cells in skeletal-muscle^(65)^ | *Brain processes*  ▪ EP1 receptor activation increases the probability of status epilepticus and independently promotes hippocampal neurodegeneration^(80)^  ▪ Behavioral alterations (social avoidance) caused by repeated stress^(81, 82)^  Nervous system  ▪ Pain perception^(47)^ |
| *Gastro-intestinal*  ▪ Inhibition of stomach acid secretion^(83)^  ▪ Bicarbonate secretion in stomach and duodenum^(83)^ | *Gastro-intestinal*  ▪ Stimulating mucus secretion^(83)^  ▪ Bicarbonate secretion in the duodenum^(83)^ | *Gastro-intestinal* | *Gastro-intestinal*  ▪ functions as a constrictor in the smooth muscles of the gastrointestinal tract  ▪ Inhibition of gastric motility^(83)^  ▪ Bicarbonate secretion in the stomach^(83)^  ▪ Cytoprotection in the stomach^(84)^ |
| Bone, cartilage, muscles  ▪ Contraction of uterus: dysmenorrhoea and parturition | Bone, cartilage, muscles  ▪ Cell-proliferation for muscle and bone and other tissue repair  ▪ osteoarthritis^(85, 86)^ | Bone, cartilage, muscles  ▪ Apoptosis of neuronal cells in skeletal-muscle^(65)^  ▪ Myalgia^(27, 87)^ | Bone, cartilage, muscles |
| *Cardiovascular*  ▪ Reduced contractility of the heart^(88)^  ▪ cardiac hypertrophy^(89)^  ▪ Coagulation thrombosis^(40)^ | *Cardiovascular*  ▪ Endothelial barrier integrity^(90, 91) (92)^  ▪ Inflammatory reaction in atherosclerotic plaques^(93)^ | *Cardiovascular* | *Cardiovascular* |
| *Lungs*  ▪ Bronchoconstriction  /Cough^(7, 94)^ | *Lungs*  ▪ Bronchodilation  ▪ a key role for EP2 and EP4 receptors in microvascular leak (MVL) induced by PGE2^(36)^ | *Lungs*  ▪ Inhibition of bronchoconstriction^(95)^  Suppresses lung innate immunity^(96)^  ▪ Suppresses allergic sensitization and lung-inflammation^(97)^ | *Lungs*  ▪ functions as a constrictor in the smooth muscles of the trachea |
| *Kidneys*  Downregulates COX2  Attenuates reabsorption  Water retention under high salt conditions^(98)^  Edema^(99)^  Role in AKI^(34)^  Role in polyuria^(39)^ | *Kidneys* | *Kidneys* | *Kidneys*  ▪ Glomerulosclerosis^(100)^  ▪ Main role in renal fibrosis^(101)^ |
| *Skin*  ▪ Stimulates melanocyte dendricity, skin pigmentation (also through) EP1^(102)^ | *Skin* | *Skin* | *Skin* |
| *Antagonizing other hormones*  ▪ Inhibition of serotonin signaling; inflammatory pain^(103)^  ▪ Inhibition of release (NOS) of noradrenaline and serotonin and dopamine^(104-106)^ |  |  |  |

**References**

1. Herlenius E. An inflammatory pathway to apnea and autonomic dysregulation. Respir Physiol Neurobiol. 2011;178(3):449-57. DOI: [10.1016/j.resp.2011.06.026](https://doi.org/10.1016/j.resp.2011.06.026)

2. Lang F, Abed M, Lang E, Föller M. Oxidative stress and suicidal erythrocyte death. Antioxid Redox Signal. 2014;21(1):138-53. DOI: [10.1089/ars.2013.5747](https://doi.org/10.1089/ars.2013.5747)

3. Wang W, Liang M, Wang L, Bei W, Rong X, Xu J, et al. Role of prostaglandin E2 in macrophage polarization: Insights into atherosclerosis. Biochem Pharmacol. 2023;207:115357. DOI: [10.1016/j.bcp.2022.115357](https://doi.org/10.1016/j.bcp.2022.115357)

4. Machado-Carvalho L, Roca-Ferrer J, Picado C. Prostaglandin E2 receptors in asthma and in chronic rhinosinusitis/nasal polyps with and without aspirin hypersensitivity. Respir Res. 2014;15(1):100. DOI: [10.1186/s12931-014-0100-7](https://doi.org/10.1186/s12931-014-0100-7)

5. Kadoyama K, Takahashi Y, Higashida H, Tanabe T, Yoshimoto T. Overexpression of cyclooxygenase-2 stimulates amyloid β-peptide production in neuronal cells. International Congress Series. 2002;1233:349-53. DOI: [10.1006/bbrc.2001.4357](https://doi.org/10.1006/bbrc.2001.4357)

6. Z. H. Zhang, Y. Yu, S. G. Wei, Y. Nakamura, K. Nakamura and R. B. Felder. EP₃ receptors mediate PGE₂-induced hypothalamic paraventricular nucleus excitation and sympathetic activation. Am J Physiol Heart Circ Physiol 2011 Vol. 301 Issue 4 Pages H1559-69.
DOI: [10.1152/ajpheart.00262.2011](https://doi.org/10.1152/ajpheart.00262.2011)7. Zhou J, Alvarez-Elizondo MB, Botvinick E, George SC. Adenosine A(1) and prostaglandin E receptor 3 receptors mediate global airway contraction after local epithelial injury. Am J Respir Cell Mol Biol. 2013;48(3):299-305. DOI: [10.1165/rcmb.2012-0174OC](https://doi.org/10.1165/rcmb.2012-0174oc)8. Sun X, Li Q. Prostaglandin EP2 receptor: Novel therapeutic target for human cancers (Review). Int J Mol Med. 2018;42(3):1203-14. DOI: [10.3892/ijmm.2018.3744](https://doi.org/10.3892/ijmm.2018.3744)9. Maher SA, Birrell MA, Belvisi MG. Prostaglandin E2 mediates cough via the EP3 receptor: implications for future disease therapy. Am J Respir Crit Care Med. 2009;180(10):923-8. DOI: [10.1164/rccm.200903-0388OC](https://doi.org/10.1164/rccm.200903-0388oc)10. Meyer-Kirchrath J, Martin M, Schooss C, Jacoby C, Flögel U, Marzoll A, et al. Overexpression of prostaglandin EP3 receptors activates calcineurin and promotes hypertrophy in the murine heart. Cardiovasc Res. 2009;81(2):310-8.
DOI: [10.1093/cvr/cvn312](https://doi.org/10.1093/cvr/cvn312)

11. Bryson TD, Harding P. Prostaglandin E2 EP receptors in cardiovascular disease: An update. Biochemical Pharmacology. 2022;195:114858. <https://doi.org/10.1016/j.bcp.2021.114858>

12. Amior L, Srivastava R, Nano R, Bertuzzi F, Melloul D. The role of Cox-2 and prostaglandin E2 receptor EP3 in pancreatic β-cell death. The FASEB Journal. 2019;33(4):4975-86. DOI: [10.1096/fj.201801823R](https://doi.org/10.1096/fj.201801823r)

13. Wang W, Zhong X, Guo J. Role of 2‑series prostaglandins in the pathogenesis of type 2 diabetes mellitus and non‑alcoholic fatty liver disease (Review). Int J Mol Med. 2021;47(6):114. DOI: [10.3892/ijmm.2021.4947](https://doi.org/10.3892/ijmm.2021.4947)

14. Downie J, Poyser NL, Wunderlich M. Levels of prostaglandins in human endometrium during the normal menstrual cycle. J Physiol. 1974;236(2):465-72.
DOI: [10.1113/jphysiol.1974.sp010446](https://doi.org/10.1113/jphysiol.1974.sp010446)

15. Lieb J, Karmali R, Horrobin D. Elevated levels of prostaglandin E2 and thromboxane B2 in depression. Prostaglandins Leukot Med. 1983;10(4):361-7.
DOI: [10.1016/0262-1746(83)90048-3](https://doi.org/10.1016/0262-1746(83)90048-3)

16. Yin J, Xia W, Li Y, Guo C, Zhang Y, Huang S, et al. COX-2 mediates PM2.5-induced apoptosis and inflammation in vascular endothelial cells. Am J Transl Res. 2017;9(9):3967-76. PMID: [28979673](https://pubmed.ncbi.nlm.nih.gov/28979673/)

17. Takafuji V, Cosme R, Lublin D, Lynch K, Roche JK. Prostanoid receptors in intestinal epithelium: selective expression, function, and change with inflammation. Prostaglandins Leukot Essent Fatty Acids. 2000;63(4):223-35. DOI: [10.1054/plef.2000.0144](https://doi.org/10.1054/plef.2000.0144)

18. Grabauskas G, Wu X, Gao J, Li J-Y, Turgeon DK, Owyang C. Prostaglandin E<sub>2</sub>, Produced by Mast Cells in Colon Tissues From Patients With Irritable Bowel Syndrome, Contributes to Visceral Hypersensitivity in Mice. Gastroenterology. 2020;158(8):2195-207.e6. DOI: [10.1053/j.gastro.2020.02.022](https://doi.org/10.1053/j.gastro.2020.02.022)

19. Yang T, Du Y. Distinct roles of central and peripheral prostaglandin E2 and EP subtypes in blood pressure regulation. Am J Hypertens. 2012;25(10):1042-9.
DOI: [10.1038/ajh.2012.67](https://doi.org/10.1038/ajh.2012.67)

20. Gupta A, Chander Chiang K. Prostaglandin D(2) as a mediator of lymphopenia and a therapeutic target in COVID-19 disease. Med Hypotheses. 2020;143:110122.
DOI: [10.1016/j.mehy.2020.110122](https://doi.org/10.1016/j.mehy.2020.110122)

21. Fritz M, Klawonn AM, Nilsson A, Singh AK, Zajdel J, Wilhelms DB, et al. Prostaglandin-dependent modulation of dopaminergic neurotransmission elicits inflammation-induced aversion in mice. J Clin Invest. 2016;126(2):695-705. DOI: [10.1172/JCI83844](https://doi.org/10.1172/jci83844)

22. Antonova M. Prostaglandins and prostaglandin receptor antagonism in migraine. Dan Med J. 2013;60(5):B4635. **PMCID: PMC3619997**

23. Chen J, Deng JC, Zemans RL, Bahmed K, Kosmider B, Zhang M, et al. Age-induced prostaglandin E(2) impairs mitochondrial fitness and increases mortality to influenza infection. Nat Commun. 2022;13(1):6759. <https://doi.org/10.1038/s41467-022-34593-y>

24. Bryson TD, Zurek M, Moore C, Taube D, Datta I, Levin A, et al. Prostaglandin E2 affects mitochondrial function in adult mouse cardiomyocytes and hearts. Prostaglandins Leukot Essent Fatty Acids. 2024;201:102614. DOI: [10.1016/j.plefa.2024.102614](https://doi.org/10.1016/j.plefa.2024.102614)

25. Franco F, Wenes M, Ho P-C. Sparks Fly in PGE2-Modulated Macrophage Polarization. Immunity. 2018;49(6):987-9. DOI: [10.1016/j.immuni.2018.12.002](https://doi.org/10.1016/j.immuni.2018.12.002)

26. Liu SZ, Jemiolo B, Lavin KM, Lester BE, Trappe SW, Trappe TA. Prostaglandin E2/cyclooxygenase pathway in human skeletal muscle: influence of muscle fiber type and age. J Appl Physiol (1985). 2016;120(5):546-51. DOI: [10.1152/japplphysiol.00396.2015](https://doi.org/10.1152/japplphysiol.00396.2015)

27. Hedenberg-Magnusson B, Ernberg M, Alstergren P, Kopp S. Pain mediation by prostaglandin E2 and leukotriene B4 in the human masseter muscle. Acta Odontol Scand. 2001;59(6):348-55. DOI: [10.1080/000163501317153185](https://doi.org/10.1080/000163501317153185)

28. Fain JN, Ballou LR, Bahouth SW. Obesity is induced in mice heterozygous for cyclooxygenase-2. Prostaglandins Other Lipid Mediat. 2001;65(4):199-209.
DOI: [10.1016/s0090-6980(01)00136-8](https://doi.org/10.1016/s0090-6980(01)00136-8)

29. Gadsby R, Barnie-Adshead A, Grammatoppoulos D, Gadsby P. Nausea and vomiting in pregnancy: an association between symptoms and maternal prostaglandin E2. Gynecol Obstet Invest. 2000;50(3):149-52. DOI: [10.1159/000010314](https://doi.org/10.1159/000010314)

30. Milatovic D, Montine TJ, Aschner M. Prostanoid signaling: dual role for prostaglandin E2 in neurotoxicity. Neurotoxicology. 2011;32(3):312-9. DOI: [10.1016/j.neuro.2011.02.004](https://doi.org/10.1016/j.neuro.2011.02.004)

31. Yang D, Xu K, Xu X, Xu P. Revisiting prostaglandin E2: A promising therapeutic target for osteoarthritis. Clin Immunol. 2024;260:109904. DOI: [10.1016/j.clim.2024.109904](https://doi.org/10.1016/j.clim.2024.109904)

32. Kawabata A. Prostaglandin E2 and pain--an update. Biol Pharm Bull. 2011;34(8):1170-3. DOI: [10.1248/bpb.34.1170](https://doi.org/10.1248/bpb.34.1170)

33. Ikeda-Matsuo Y, Miyata H, Mizoguchi T, Ohama E, Naito Y, Uematsu S, et al. Microsomal prostaglandin E synthase-1 is a critical factor in dopaminergic neurodegeneration in Parkinson's disease. Neurobiology of Disease. 2019;124:81-92.
DOI: [10.1016/j.nbd.2018.11.004](https://doi.org/10.1016/j.nbd.2018.11.004)

34. Leng J, Zhao W, Guo J, Yu G, Zhu G, Ge J, et al. E-prostanoid 3 receptor deficiency on myeloid cells protects against ischemic acute kidney injury via breaking the auto-amplification loop of necroinflammation. Kidney International. 2023;103(1):100-14.
DOI: [10.1016/j.kint.2022.08.019](https://doi.org/10.1016/j.kint.2022.08.019)

35. Jia Z, Zhang Y, Ding G, Heiney KM, Huang S, Zhang A. Role of COX-2/mPGES-1/prostaglandin E2 cascade in kidney injury. Mediators Inflamm. 2015;2015:147894.
DOI: [10.1155/2015/147894](https://doi.org/10.1155/2015/147894)

36. Jones VC, Birrell MA, Maher SA, Griffiths M, Grace M, O'Donnell VB, et al. Role of EP2 and EP4 receptors in airway microvascular leak induced by prostaglandin E2. British journal of pharmacology. 2016;173(6):992-1004. DOI: [10.1111/bph.13400](https://doi.org/10.1111/bph.13400)

37. Machado-Carvalho L, Roca-Ferrer J, Picado C. Prostaglandin E2 receptors in asthma and in chronic rhinosinusitis/nasal polyps with and without aspirin hypersensitivity. Respiratory Research. 2014;15(1):100. DOI: [10.1186/s12931-014-0100-7](https://doi.org/10.1186/s12931-014-0100-7)

38. Huang ZL, Urade Y, Hayaishi O. Prostaglandins and adenosine in the regulation of sleep and wakefulness. Curr Opin Pharmacol. 2007;7(1):33-8.
DOI: [10.1016/j.coph.2006.09.004](https://doi.org/10.1016/j.coph.2006.09.004)

39. Hassouneh R, Nasrallah R, Zimpelmann J, Gutsol A, Eckert D, Ghossein J, et al. PGE2 receptor EP3 inhibits water reabsorption and contributes to polyuria and kidney injury in a streptozotocin-induced mouse model of diabetes. Diabetologia. 2016;59(6):1318-28.
DOI: [10.1007/s00125-016-3916-5](https://doi.org/10.1007/s00125-016-3916-5)

40. Mawhin MA, Tilly P, Fabre JE. The receptor EP3 to PGE2: A rational target to prevent atherothrombosis without inducing bleeding. Prostaglandins Other Lipid Mediat. 2015 Sep;121(Pt A):4-16. DOI: [10.1016/j.prostaglandins.2015.10.001](https://doi.org/10.1016/j.prostaglandins.2015.10.001)

41. Biringer RG. A Review of Prostanoid Receptors: Expression, Characterization, Regulation, and Mechanism of Action. J Cell Commun Signal. 2021;15(2):155-84.
DOI: [10.1007/s12079-020-00585-0](https://doi.org/10.1007/s12079-020-00585-0)

42. Markovič T, Jakopin Ž, Dolenc MS, Mlinarič-Raščan I. Structural features of subtype-selective EP receptor modulators. Drug Discovery Today. 2017;22(1):57-71.
DOI: [10.1016/j.drudis.2016.08.003](https://doi.org/10.1016/j.drudis.2016.08.003)

43. Kawahara K, Hohjoh H, Inazumi T, Tsuchiya S, Sugimoto Y. Prostaglandin E2-induced inflammation: Relevance of prostaglandin E receptors. Biochim Biophys Acta. 2015;1851(4):414-21. DOI: [10.1016/j.bbalip.2014.07.008](https://doi.org/10.1016/j.bbalip.2014.07.008)

44. Regan JW, Bailey TJ, Donello JE, Pierce KL, Pepperl DJ, Zhang D, et al. Molecular cloning and expression of human EP3 receptors: evidence of three variants with differing carboxyl termini. Br J Pharmacol. 1994;112(2):377-85.
DOI: [10.1111/j.1476-5381.1994.tb13082.x](https://doi.org/10.1111/j.1476-5381.1994.tb13082.x)

45. Israel DD, Regan JW. EP(3) prostanoid receptor isoforms utilize distinct mechanisms to regulate ERK 1/2 activation. Biochim Biophys Acta. 2009;1791(4):238-45.
DOI: [10.1016/j.bbalip.2009.01.021](https://doi.org/10.1016/j.bbalip.2009.01.021)

46. Wang L, Wu Y, Jia Z, Yu J, Huang S. Roles of EP Receptors in the Regulation of Fluid Balance and Blood Pressure. Front Endocrinol (Lausanne). 2022;13:875425.
DOI: [10.3389/fendo.2022.875425](https://doi.org/10.3389/fendo.2022.875425)

47. Williams JA, Shacter E. Regulation of macrophage cytokine production by prostaglandin E2. Distinct roles of cyclooxygenase-1 and -2. J Biol Chem. 1997 Oct 10;272(41):25693-9. DOI: [10.1074/jbc.272.41.25693](https://doi.org/10.1074/jbc.272.41.25693)

48. HyeonJoo Cheon, Young Hee Rho, Seong Jae Choi, Young Ho Lee, Gwan Gyu Song, Jeongwon Sohn, Nam Hee Won, Jong Dae Ji; Prostaglandin E_2_ Augments IL-10 Signaling and Function[^1^](about:blank). *J Immunol* 15 July 2006; 177 (2): 1092–1100. <https://doi.org/10.4049/jimmunol.177.2.1092>

49. Nam J, Kwon B, Yoon Y, Choe J. PGE2 stimulates COX-2 expression via EP2/4 receptors and acts in synergy with IL-1&#x3b2; in human follicular dendritic cell-like cells. European Journal of Inflammation. 2018;16. DOI:[10.1177/2058739218796386](https://doi.org/10.1177/2058739218796386)

50. Tsuge K, Inazumi T, Shimamoto A, Sugimoto Y. Molecular mechanisms underlying prostaglandin E2-exacerbated inflammation and immune diseases. Int Immunol. 2019;31(9):597-606. DOI: [10.1093/intimm/dxz021](https://doi.org/10.1093/intimm/dxz021)

51. Vio CP, Quiroz-Munoz M, Cuevas CA, Cespedes C, Ferreri NR. Prostaglandin E2 EP3 receptor regulates cyclooxygenase-2 expression in the kidney. Am J Physiol Renal Physiol. 2012;303(3):F449-57. DOI: [10.1152/ajprenal.00634.2011](https://doi.org/10.1152/ajprenal.00634.2011)

52. Haddad A, Flint-Ashtamker G, Minzel W, Sood R, Rimon G, Barki-Harrington L. Prostaglandin EP1 Receptor Down-regulates Expression of Cyclooxygenase-2 by Facilitating Its Proteasomal Degradation. Journal of Biological Chemistry. 2012;287(21):17214-23.
DOI: [10.1074/jbc.M111.304220](https://doi.org/10.1074/jbc.m111.304220)

53. U. R. Mbonye, C. Yuan, C. E. Harris, R. S. Sidhu, I. Song, T. Arakawa, et al. Two Distinct Pathways for Cyclooxygenase-2 Protein Degradation. Journal of Biological Chemistry 2008 Vol. 283 Issue 13 Pages 8611-8623 DOI: [10.1074/jbc.M710137200](https://doi.org/10.1074/jbc.M710137200)54. Fang KM, Shu WH, Chang HC, Wang JJ, Mak OT. Study of prostaglandin receptors in mitochondria on apoptosis of human lung carcinoma cell line A549. Biochem Soc Trans. 2004;32(Pt 6):1078-80. DOI: [10.1042/BST0321078](https://doi.org/10.1042/bst0321078)55. Bhattacharya M, Peri K, Ribeiro-da-Silva A, Almazan G, Shichi H, Hou X, et al. Localization of functional prostaglandin E2 receptors EP3 and EP4 in the nuclear envelope. J Biol Chem. 1999;274(22):15719-24. DOI: [10.1074/jbc.274.22.15719](https://doi.org/10.1074/jbc.274.22.15719)56. Kimple ME, Keller MP, Rabaglia MR, Pasker RL, Neuman JC, Truchan NA, et al. Prostaglandin E2 receptor, EP3, is induced in diabetic islets and negatively regulates glucose- and hormone-stimulated insulin secretion. Diabetes. 2013;62(6):1904-12.
DOI: [10.2337/db12-0769](https://doi.org/10.2337/db12-0769)

57. Strong P, Coleman RA, Humphrey PP. Prostanoid-induced inhibition of lipolysis in rat isolated adipocytes: probable involvement of EP3 receptors. Prostaglandins. 1992 Jun;43(6):559-66. DOI: [10.1016/0090-6980(92)90115-a](https://doi.org/10.1016/0090-6980(92)90115-a)

58. Ceddia RP, Lee D, Maulis MF, Carboneau BA, Threadgill DW, Poffenberger G, et al. The PGE2 EP3 Receptor Regulates Diet-Induced Adiposity in Male Mice. Endocrinology. 2016;157(1):220-32. DOI: [10.1210/en.2015-1693](https://doi.org/10.1210/en.2015-1693)

59. Xu H, Fu JL, Miao YF, Wang CJ, Han QF, Li S, et al. Prostaglandin E2 receptor EP3 regulates both adipogenesis and lipolysis in mouse white adipose tissue. J Mol Cell Biol. 2016;8(6):518-29. DOI: [10.1093/jmcb/mjw035](https://doi.org/10.1093/jmcb/mjw035)

60. Püschel GP, Kirchner C, Schröder A, Jungermann K. Glycogenolytic and antiglycogenolytic prostaglandin E2 actions in rat hepatocytes are mediated via different signalling pathways. Eur J Biochem. 1993;218(3):1083-9.
DOI: [10.1111/j.1432-1033.1993.tb18468.x](https://doi.org/10.1111/j.1432-1033.1993.tb18468.x)

61. Carboneau BA, Allan JA, Townsend SE, Kimple ME, Breyer RM, Gannon M. Opposing effects of prostaglandin E2 receptors EP3 and EP4 on mouse and human β-cell survival and proliferation. Molecular Metabolism. 2017;6(6):548-59. DOI: [10.1016/j.molmet.2017.04.002](https://doi.org/10.1016/j.molmet.2017.04.002)

62. Ying F, Cai Y, Wong HK, Chen XY, Huang IB, Vanhoutte PM, et al. EP4 emerges as a novel regulator of bile acid synthesis and its activation protects against hypercholesterolemia. Biochimica et Biophysica Acta (BBA) - Molecular and Cell Biology of Lipids. 2018;1863(9):1029-40. DOI: [10.1016/j.bbalip.2018.06.003](https://doi.org/10.1016/j.bbalip.2018.06.003)

63. Liu J, He H, Zhang Z, Jiang S, Akahoshi T, Yang J, et al. Mitochondria play a role in the development of non-apoptotic programmed cell death of neutrophils induced by ONO-AE-248. Cell Mol Immunol. 2007;4(6):447-53. PMID: **18163956**

64. Palumbo S, Toscano CD, Parente L, Weigert R, Bosetti F. The cyclooxygenase-2 pathway via the PGE₂ EP2 receptor contributes to oligodendrocytes apoptosis in cuprizone-induced demyelination. J Neurochem. 2012;121(3):418-27.
DOI: [10.1111/j.1471-4159.2011.07363.x](https://doi.org/10.1111/j.1471-4159.2011.07363.x)

65. Kosuge Y, Nango H, Kasai H, Yanagi T, Mawatari T, Nishiyama K, et al. Generation of Cellular Reactive Oxygen Species by Activation of the EP2 Receptor Contributes to Prostaglandin E2-Induced Cytotoxicity in Motor Neuron-Like NSC-34 Cells. Oxid Med Cell Longev. 2020;2020:6101838. DOI: [10.1155/2020/6101838](https://doi.org/10.1155/2020/6101838)

66. Carrasco E, Casper D, Werner P. PGE(2) receptor EP1 renders dopaminergic neurons selectively vulnerable to low-level oxidative stress and direct PGE(2) neurotoxicity. J  Neurosci Res. 2007;85(14):3109-17. DOI: [10.1002/jnr.21425](https://doi.org/10.1002/jnr.21425)

67. Ahmad AS, Maruyama T, Narumiya S, Doré S. PGE2 EP1 receptor deletion attenuates 6-OHDA-induced Parkinsonism in mice: old switch, new target. Neurotox Res. 2013;23(3):260-6. DOI: [10.1007/s12640-013-9381-8](https://doi.org/10.1007/s12640-013-9381-8)

68. Mukai Y, Okubo TS, Lazarus M, Ono D, Tanaka KF, Yamanaka A. Prostaglandin E(2) Induces Long-Lasting Inhibition of Noradrenergic Neurons in the Locus Coeruleus and Moderates the Behavioral Response to Stressors. J Neurosci. 2023;43(47):7982-99.
DOI: [10.1523/JNEUROSCI.0353-23.2023](https://doi.org/10.1523/jneurosci.0353-23.2023)

69. Suzuki C, Miyamoto C, Furuyashiki T, Narumiya S, Ohinata K. Central PGE2 exhibits anxiolytic-like activity via EP1 and EP4 receptors in a manner dependent on serotonin 5-HT1A, dopamine D1 and GABAA receptors. FEBS Lett. 2011;585(14):2357-62.
DOI: [10.1016/j.febslet.2011.06.004](https://doi.org/10.1016/j.febslet.2011.06.004)

70. Lushington R, Camilli S, Pascual F, Lockey RF, Kolliputi N. EP2 inhibition restores myeloid metabolism and reverses cognitive decline. Journal of Allergy and Clinical Immunology: Global. 2023;2(2):100082. DOI: [10.1016/j.jacig.2023.100082](https://doi.org/10.1016/j.jacig.2023.100082)

71. Kang X, Qiu J, Li Q, Bell KA, Du Y, Jung DW, et al. Cyclooxygenase-2 contributes to oxidopamine-mediated neuronal inflammation and injury via the prostaglandin E2 receptor EP2 subtype. Scientific Reports. 2017;7(1):9459. DOI: [10.1038/s41598-017-09528-z](https://doi.org/10.1038/s41598-017-09528-z)

72. Jin J, Shie FS, Liu J, Wang Y, Davis J, Schantz AM, et al. Prostaglandin E2 receptor subtype 2 (EP2) regulates microglial activation and associated neurotoxicity induced by aggregated alpha-synuclein. J Neuroinflammation. 2007;4:2. DOI: [10.1186/1742-2094-4-2](https://doi.org/10.1186/1742-2094-4-2)

73. Hu W, Mathey E, Hartung HP, Kieseier BC. Cyclo-oxygenases and prostaglandins in acute inflammatory demyelination of the peripheral nerve. Neurology. 2003;61(12):1774-9.
DOI: [10.1212/01.wnl.0000098884.75756.4d](https://doi.org/10.1212/01.wnl.0000098884.75756.4d)

74. Kitaoka S. Microglia regulate neuronal and behavioural functions under physiological and pathological conditions. J Biochem. 2023 Mar 7;173(3):153-157. PMID: 36539335.
DOI: [10.1093/jb/mvac099](https://doi.org/10.1093/jb/mvac099)

75. Fritz M, Klawonn AM, Nilsson A, Singh AK, Zajdel J, Wilhelms DB, et al. Prostaglandin-dependent modulation of dopaminergic neurotransmission elicits inflammation-induced aversion in mice. J Clin Invest. 2016;126(2):695-705. DOI: [10.1172/JCI83844](https://doi.org/10.1172/jci83844)

76. Furuyashiki T, Narumiya S. Stress responses: the contribution of prostaglandin E(2) and its receptors. Nat Rev Endocrinol. 2011;7(3):163-75. DOI: [10.1038/nrendo.2010.194](https://doi.org/10.1038/nrendo.2010.194)

77. Taiwo Y, Levine J. Prostaglandins inhibit endogenous pain control mechanisms by blocking transmission at spinal noradrenergic synapses. The Journal of Neuroscience. 1988;8(4):1346-9. DOI: [10.1523/JNEUROSCI.08-04-01346.1988](https://doi.org/10.1523/jneurosci.08-04-01346.1988)

78. Spicuzza L, Giembycz MA, Barnes PJ, Belvisi MG. Prostaglandin E2 suppression of acetylcholine release from parasympathetic nerves innervating guinea-pig trachea by interacting with prostanoid receptors of the EP3-subtype. British Journal of Pharmacology. 1998;123(6):1246-52. DOI: [10.1038/sj.bjp.0701720](https://doi.org/10.1038/sj.bjp.0701720)

79. Nakao K, Murase A, Ohshiro H, Okumura T, Taniguchi K, Murata Y, et al. CJ-023,423, a novel, potent and selective prostaglandin EP4 receptor antagonist with antihyperalgesic properties. J Pharmacol Exp Ther. 2007;322(2):686-94. DOI: [10.1124/jpet.107.122010](https://doi.org/10.1124/jpet.107.122010)

80. Rojas A, Gueorguieva P, Lelutiu N, Quan Y, Shaw R, Dingledine R. The prostaglandin EP1 receptor potentiates kainate receptor activation via a protein kinase C pathway and exacerbates status epilepticus. Neurobiol Dis. 2014;70:74-89.
DOI: [10.1016/j.nbd.2014.06.004](https://doi.org/10.1016/j.nbd.2014.06.004)

81. Furuyashiki T. Roles of dopamine and inflammation-related molecules in behavioral alterations caused by repeated stress. J Pharmacol Sci. 2012;120(2):63-9.
DOI: [10.1254/jphs.12r09cp](https://doi.org/10.1254/jphs.12r09cp)

82. Furuyashiki T, Narumiya S. Roles of prostaglandin E receptors in stress responses. Curr Opin Pharmacol. 2009;9(1):31-8. DOI: [10.1016/j.coph.2008.12.010](https://doi.org/10.1016/j.coph.2008.12.010)

83. K. Takeuchi. Prostaglandin EP receptors and their roles in mucosal protection and ulcer healing in the gastrointestinal tract. Adv Clin Chem 2010 Vol. 51 Pages 121-44
DOI: [10.1016/s0065-2423(10)51005-9](https://doi.org/10.1016/s0065-2423(10)51005-9)

84. Araki H, Ukawa H, Sugawa Y, Yagi K, Suzuki K, Takeuchi K. The roles of prostaglandin E receptor subtypes in the cytoprotective action of prostaglandin E2 in rat stomach. Aliment Pharmacol Ther. 2000;14 Suppl 1:116-24. DOI: [10.1046/j.1365-2036.2000.014s1116.x](https://doi.org/10.1046/j.1365-2036.2000.014s1116.x)

85. Jiang W, Jin Y, Zhang S, Ding Y, Huo K, Yang J, et al. PGE2 activates EP4 in subchondral bone osteoclasts to regulate osteoarthritis. Bone Res. 2022;10(1):27.
DOI: [10.1038/s41413-022-00201-4](https://doi.org/10.1038/s41413-022-00201-4)

86. Clark P, Rowland SE, Denis D, Mathieu MC, Stocco R, Poirier H, et al. MF498 [N-{[4-(5,9-Diethoxy-6-oxo-6,8-dihydro-7H-pyrrolo[3,4-g]quinolin-7-yl)-3-methylbenzyl]sulfonyl}-2-(2-methoxyphenyl)acetamide], a selective E prostanoid receptor 4 antagonist, relieves joint inflammation and pain in rodent models of rheumatoid and osteoarthritis. J Pharmacol Exp Ther. 2008;325(2):425-34. DOI: [10.1124/jpet.107.134510](https://doi.org/10.1124/jpet.107.134510)

87. Hedenberg-Magnusson B, Ernberg M, Alstergren P, Kopp S. Effect on prostaglandin E2 and leukotriene B4 levels by local administration of glucocorticoid in human masseter muscle myalgia. Acta Odontol Scand. 2002;60(1):29-36.
DOI: [10.1080/000163502753471970](https://doi.org/10.1080/000163502753471970)

88. Gu X, Xu J, Zhu L, Bryson T, Yang XP, Peterson E, et al. Prostaglandin E2 Reduces Cardiac Contractility via EP3 Receptor. Circ Heart Fail. 2016;9(8).
DOI: [10.1161/CIRCHEARTFAILURE.116.003291](https://doi.org/10.1161/circheartfailure.116.003291)

89. Maxwell DL, Bryson TD, Taube D, Xu J, Peterson E, Harding P. Deleterious effects of cardiomyocyte-specific prostaglandin E2 EP3 receptor overexpression on cardiac function after myocardial infarction. Life Sci. 2023;313:121277. DOI: [10.1016/j.lfs.2022.121277](https://doi.org/10.1016/j.lfs.2022.121277)

90. Bärnthaler T, Maric J, Platzer W, Konya V, Theiler A, Hasenöhrl C, et al. The Role of PGE(2) in Alveolar Epithelial and Lung Microvascular Endothelial Crosstalk. Sci Rep. 2017;7(1):7923. DOI: [10.1038/s41598-017-08228-y](https://doi.org/10.1038/s41598-017-08228-y)

91. Theiler A, Konya V, Pasterk L, Maric J, Bärnthaler T, Lanz I, et al. The EP1/EP3 receptor agonist 17-pt-PGE2 acts as an EP4 receptor agonist on endothelial barrier function and in a model of LPS-induced pulmonary inflammation. Vascular Pharmacology. 2016;87:180-9. 
DOI: [10.1016/j.vph.2016.09.008](https://doi.org/10.1016/j.vph.2016.09.008)

92. Perrot CY, Herrera JL, Fournier-Goss AE, Komatsu M. Prostaglandin E2 breaks down pericyte-endothelial cell interaction via EP1 and EP4-dependent downregulation of pericyte N-cadherin, connexin-43, and R-Ras. Sci Rep. 2020;10(1):11186.
DOI: [10.1038/s41598-020-68019-w](https://doi.org/10.1038/s41598-020-68019-w)

93. Cipollone F, Fazia ML, Iezzi A, Cuccurullo C, De Cesare D, Ucchino S, et al. Association between prostaglandin E receptor subtype EP4 overexpression and unstable phenotype in atherosclerotic plaques in human. Arterioscler Thromb Vasc Biol. 2005;25(9):1925-31. <https://doi.org/10.1161/01.ATV.0000177814.41505.41>

94. Okazaki A, Hara J, Ohkura N, Fujimura M, Sakai T, Abo M, et al. Role of prostaglandin E(2) in bronchoconstriction-triggered cough response in guinea pigs. Pulm Pharmacol Ther. 2018;48:62-70. DOI: [10.1016/j.pupt.2017.09.003](https://doi.org/10.1016/j.pupt.2017.09.003)

95. Säfholm J, Manson ML, Bood J, Delin I, Orre AC, Bergman P, et al. Prostaglandin E2 inhibits mast cell-dependent bronchoconstriction in human small airways through the E prostanoid subtype 2 receptor. J Allergy Clin Immunol. 2015;136(5):1232-9.e1.
DOI: [10.1016/j.jaci.2015.04.002](https://doi.org/10.1016/j.jaci.2015.04.002)

96. Aronoff DM, Bergin IL, Lewis C, Goel D, O'Brien E, Peters-Golden M, et al. E-prostanoid 2 receptor signaling suppresses lung innate immunity against Streptococcus pneumoniae. Prostaglandins Other Lipid Mediat. 2012;98(1-2):23-30.
DOI: [10.1016/j.prostaglandins.2012.03.002](https://doi.org/10.1016/j.prostaglandins.2012.03.002)

97. Zasłona Z, Okunishi K, Bourdonnay E, Domingo-Gonzalez R, Moore BB, Lukacs NW, et al. Prostaglandin E₂ suppresses allergic sensitization and lung inflammation by targeting the E prostanoid 2 receptor on T cells. J Allergy Clin Immunol. 2014;133(2):379-87. 
DOI: [10.1016/j.jaci.2013.07.037](https://doi.org/10.1016/j.jaci.2013.07.037)

98. Xiao L, Itani HA, Carmo LSd, Carver LS, Breyer RM, Harrison DG. Central EP3 (E Prostanoid 3) Receptors Mediate Salt-Sensitive Hypertension and Immune Activation. Hypertension. 2019;74(6):1507-15. DOI: [10.1161/HYPERTENSIONAHA.119.13850](https://doi.org/10.1161/hypertensionaha.119.13850)

99. Morimoto K, Shirata N, Taketomi Y, Tsuchiya S, Segi-Nishida E, Inazumi T, et al. Prostaglandin E2–EP3 Signaling Induces Inflammatory Swelling by Mast Cell Activation. The Journal of Immunology. 2014;192(3):1130-7. DOI: [10.4049/jimmunol.1300290](https://doi.org/10.4049/jimmunol.1300290)

100. Chen X, Yin J, Xu Y, Qiu Z, Liu J, Chen X. Effect of selective inhibition or activation of PGE2 EP1 receptor on glomerulosclerosis. Mol Med Rep. 2020;22(4):2887-95.
DOI: [10.3892/mmr.2020.11353](https://doi.org/10.3892/mmr.2020.11353)

101. Kresse JC, Mutsaers HAM, Jensen MS, Tingskov SJ, Madsen MG, Nejsum LN, et al. EP(1) receptor antagonism mitigates early and latestage renal fibrosis. Acta Physiol (Oxf). 2022;234(3):e13780. DOI: [10.1111/apha.13780](https://doi.org/10.1111/apha.13780)

102. Scott G, Leopardi S, Printup S, Malhi N, Seiberg M, Lapoint R. Proteinase-activated receptor-2 stimulates prostaglandin production in keratinocytes: analysis of prostaglandin receptors on human melanocytes and effects of PGE2 and PGF2alpha on melanocyte dendricity. J Invest Dermatol. 2004;122(5):1214-24. DOI: [10.1111/j.0022-202X.2004.22516.x](https://doi.org/10.1111/j.0022-202x.2004.22516.x)

103. Singh AK, Zajdel J, Mirrasekhian E, Almoosawi N, Frisch I, Klawonn AM, et al. Prostaglandin-mediated inhibition of serotonin signaling controls the affective component of inflammatory pain. J Clin Invest. 2017;127(4):1370-4. DOI: [10.1172/JCI90678](https://doi.org/10.1172/jci90678)

104. al-Zadjali KH, Imler MP, Ohia SE. Inhibitory effect of prostaglandins on dopamine release from the retina. Gen Pharmacol. 1994;25(2):289-96.
DOI: [10.1016/0306-3623(94)90057-4](https://doi.org/10.1016/0306-3623(94)90057-4)

105. Günther J, Schulte K, Wenzel D, Malinowska B, Schlicker E. Prostaglandins of the E series inhibit monoamine release via EP3 receptors: proof with the competitive EP3 receptor antagonist L-826,266. Naunyn Schmiedebergs Arch Pharmacol. 2010;381(1):21-31.
DOI: [10.1007/s00210-009-0478-9](https://doi.org/10.1007/s00210-009-0478-9)

106. Nakamura K, Katoh H, Ichikawa A, Negishi M. Inhibition of dopamine release by prostaglandin EP3 receptor via pertussis toxin-sensitive and -insensitive pathways in PC12 cells. J Neurochem. 1998;71(2):646-52. DOI: [10.1046/j.1471-4159.1998.71020646.x](https://doi.org/10.1046/j.1471-4159.1998.71020646.x)

(106)
