# Supplementary material for: Persistent prostaglandin E2 upregulation and hormonal multi-resistance: A hypothesis for long COVID
Source: Biochem Biophys Rep. 2026 Mar 10;46:102518. doi: 10.1016/j.bbrep.2026.102518 (PMC12994086; doi:10.1016/j.bbrep.2026.102518)
Supplement: Multimedia component 2 [file mmc2.docx]

Multimedia component 2. Hypothetical models of hormonal resistance to AVP, insulin, leptin, FGF23, IFN-gamma and serotonin

**1. Clarification of hypothetical hormonal resistance loops**

According to our hypothesis, in addition to AVP, insulin, and leptin, other PGE-stimulating hormones and agents may exhibit markedly reduced responsiveness under conditions of PGE2 dominance. Examples are:

1.1. AVP resistance

When sufficiently upregulated, PGE2 has been reported to antagonize the activity of arginine vasopressin (AVP) [^1^](#_ENREF_1). AVP, in turn, stimulates PGE2 production via its receptor V1 [^2^](#_ENREF_2). PGE2 increases osmolality through EP3 signaling [^3^](#_ENREF_3) and lowers blood pressure through EP4 and EP2 signaling [^4^](#_ENREF_4). Thus, PGE2 could cause AVP resistance in the following way: AVP is stimulated by hypovolemia when osmolality is high or when blood pressure is low. Hypovolemia induces AVP upregulation. However, under certain conditions, elevated PGE2 may dominate AVP activity, a state we term “PGE2 dominance”. PGE2 may prevent AVP from binding to its receptors by stimulating autoantibodies against AVP, as observed in another context [^5^](#_ENREF_5). Polyuria resulting from AVP malfunction can cause low blood pressure and high osmolality. In this situation, increasing AVP levels cannot restore blood pressure or osmolality. Homeostatic imbalances will continue to stimulate AVP and PGE2 levels. Moreover, AVP itself stimulates PGE2, which, due to imbalanced EP3 expression, can either raise osmolality or lower blood pressure. Thus, both AVP and PGE2 may remain upregulated in a vicious loop. See main text, Figure 1, section 6.1.

1.2. Insulin

PGE2 can increase blood glucose by inhibiting insulin secretion through EP3 signaling [^6^](#_ENREF_6), but it can also lower blood glucose by attenuating glycogenolysis through EP3 [^7^](#_ENREF_7). Either insufficient or enhanced EP3 capacity could lead to elevated glucose levels when stress factors upregulate PGE2. Elevated glucose levels, in turn, upregulate PGE2 [^8^](#_ENREF_8). We propose that PGE2 antagonizes insulin receptor binding by stimulating phosphorylation of insulin receptors (See section 5). When insulin cannot exert its function, glucose levels remain high, which in turn sustains PGE2 upregulation. In this way, glucose levels could remain elevated in both category A (EP3-up) and category B (“EP3 down”) individuals, albeit through different pathways, while PGE2 continues to antagonize insulin activity (See Figure 1).

1.3. Leptin

As discussed in the previous section, either insufficient or enhanced EP3 signaling may lead to elevated glucose levels. Leptin, which is produced in adipose tissue, is upregulated by glucose and carbohydrate intake. Leptin has been repeatedly reported to stimulate PGE2 [^9^](#_ENREF_9). When PGE2 becomes sufficiently upregulated by various factors, it may antagonize leptin activity. Under conditions of PGE2 dominance, leptin receptor binding is impaired, resulting in leptin insensitivity. This leads to increased carbohydrate intake and adipose tissue accumulation, which, under inflammatory conditions, further stimulate PGE2 production. Chronically elevated glucose levels perpetuate leptin upregulation, but leptin remains functionally antagonized because high osmolality, adipose tissue, and leptin itself all maintain elevated PGE2 levels that block leptin activity
(See main text, Figure 1).

1.4. Fibroblast growth factor 23 (FGF23)

One of the functions of fibroblast growth factor 23 (FGF23) is to promote phosphate excretion. Elevated phosphate levels have been reported in patients with long COVID patients [^10^](#_ENREF_10). Inflammation has been shown to cause FGF23 resistance [^11^](#_ENREF_11), and FGF23 itself stimulates PGE2 [^12^](#_ENREF_12). Elevated PGE2 could impair FGF23 activity, for example, by stimulating autoantibodies against FGF23, as observed in other contexts [^13^](#_ENREF_13). Increased phosphate levels stimulate higher FGF23 concentrations, and FGF23 continues to upregulate PGE2, which in turn antagonizes its function. This may result in sustained elevation of phosphate, FGF23, and PGE2 levels.

1.5. Cytokine interferon-gamma (IFN-γ)

Patients who develop long COVID often show marked upregulation of specific cytokines, including interferon-gamma (IFN-γ) [^14^](#_ENREF_14). Elevated IFN-γ can have detrimental effects [^15^](#_ENREF_15), and IFN-γ resistance has been reported in other contexts [^16^](#_ENREF_16). IFN-γ stimulates COX-2/PGE2 production [^17^](#_ENREF_17). According to our hypothesis, sufficiently high PGE2 levels may antagonize IFN-γ activity, keeping IFN-γ chronically elevated while PGE2 remains upregulated, possibly by stimulating autoantibodies. Autoantibodies against IFN-γ that block receptor binding have indeed been observed in other contexts [^18^](#_ENREF_18)^,^[^19^](#_ENREF_19).

1.6. Serotonin (5-HT)

Could a state of serotonin resistance underlie depressive symptoms? Research shows that depressed mood correlates with reduced serotonin (5-HT) activity, yet measured serotonin levels in the brains of patients with major depression are often not lower than average [^20^](#_ENREF_20). No clear explanation for this discrepancy has been established [^20^](#_ENREF_20). Serotonin has been reported to stimulate arachidonic acid (AA)COX-2/PGE2 production in the brain [^21^](#_ENREF_21). PGE2 in turn, antagonizes serotonin in several ways [^22^](#_ENREF_22) [^23^](#_ENREF_23), possibly by shared signaling pathways (section 5.1) and/or by stimulating autoantibodies to serotonin, which have been observed in the brains of psychiatric patients [^24^](#_ENREF_24). If serotonin’s receptors are fully impaired by auto antibodies, the effect would be functionally equivalent to complete serotonin deficiency, despite normal or even high levels. This mechanism may help explain the occurrence of treatment-resistant depression, as supplemented serotonin may be unable to exert its effects when its receptors are chronically blocked.

References

1 Hébert, R. L., Jacobson, H. R. & Breyer, M. D. PGE2 inhibits AVP-induced water flow in cortical collecting ducts by protein kinase C activation. *Am J Physiol* **259**, F318-325 (1990). <https://doi.org/10.1152/ajprenal.1990.259.2.F318>

2 Wuthrich, R. P. & Vallotton, M. B. Prostaglandin E2 and cyclic AMP response to vasopressin in renal medullary tubular cells. *Am J Physiol* **251**, F499-505 (1986). <https://doi.org/10.1152/ajprenal.1986.251.3.F499>

3 Hassouneh, R. *et al.* PGE2 receptor EP3 inhibits water reabsorption and contributes to polyuria and kidney injury in a streptozotocin-induced mouse model of diabetes. *Diabetologia* **59**, 1318-1328 (2016). <https://doi.org/10.1007/s00125-016-3916-5>

4 Wang, L., Wu, Y., Jia, Z., Yu, J. & Huang, S. Roles of EP Receptors in the Regulation of Fluid Balance and Blood Pressure. *Front Endocrinol (Lausanne)* **13**, 875425 (2022). <https://doi.org/10.3389/fendo.2022.875425>

5 De Bellis, A. *et al.* Detection of vasopressin cell antibodies in some patients with autoimmune endocrine diseases without overt diabetes insipidus. *Clin Endocrinol (Oxf)* **40**, 173-177 (1994). <https://doi.org/10.1111/j.1365-2265.1994.tb02464.x>

6 Kimple, M. E. *et al.* Prostaglandin E2 receptor, EP3, is induced in diabetic islets and negatively regulates glucose- and hormone-stimulated insulin secretion. *Diabetes* **62**, 1904-1912 (2013). <https://doi.org/10.2337/db12-0769>

7 Püschel, G. P., Kirchner, C., Schröder, A. & Jungermann, K. Glycogenolytic and antiglycogenolytic prostaglandin E2 actions in rat hepatocytes are mediated via different signalling pathways. *Eur J Biochem* **218**, 1083-1089 (1993). <https://doi.org/10.1111/j.1432-1033.1993.tb18468.x>

8 Sitter, T. *et al.* High glucose increases prostaglandin E2 synthesis in human peritoneal mesothelial cells: role of hyperosmolarity. *J Am Soc Nephrol* **9**, 2005-2012 (1998). <https://doi.org/10.1681/asn.V9112005>

9 Ruiz-Heiland, G., Yong, J. W., von Bremen, J. & Ruf, S. Leptin reduces in vitro cementoblast mineralization and survival as well as induces PGE2 release by ERK1/2 commitment. *Clin Oral Investig* **25**, 1933-1944 (2021). <https://doi.org/10.1007/s00784-020-03501-3>

10 Corrêa, H. L. *et al.* Phosphate and IL-10 concentration as predictors of long-covid in hemodialysis patients: A Brazilian study. *Frontiers in Immunology* **13** (2022). <https://doi.org/10.3389/fimmu.2022.1006076>

11 Rodríguez-Ortiz, M. E. *et al.* Inflammation both increases and causes resistance to FGF23 in normal and uremic rats. *Clin Sci (Lond)* **134**, 15-32 (2020). <https://doi.org/10.1042/cs20190779>

12 Syal, A. *et al.* Fibroblast growth factor-23 increases mouse PGE2 production in vivo and in vitro. *Am J Physiol Renal Physiol* **290**, F450-455 (2006). <https://doi.org/10.1152/ajprenal.00234.2005>

13 Roberts, M. S. *et al.* Autoimmune hyperphosphatemic tumoral calcinosis in a patient with FGF23 autoantibodies. *J Clin Invest* **128**, 5368-5373 (2018). <https://doi.org/10.1172/jci122004>

14 Krishna, B. A. *et al.* Spontaneous, persistent, T cell-dependent IFN-γ release in patients who progress to Long Covid. *Sci Adv* **10**, eadi9379 (2024). <https://doi.org/10.1126/sciadv.adi9379>

15 Kak, G., Raza, M. & Tiwari, B. K. Interferon-gamma (IFN-γ): Exploring its implications in infectious diseases. *Biomol Concepts* **9**, 64-79 (2018). <https://doi.org/10.1515/bmc-2018-0007>

16 Fojtova, M. *et al.* Development of IFN-γ resistance is associated with attenuation of SOCS genes induction and constitutive expression of SOCS 3 in melanoma cells. *British Journal of Cancer* **97**, 231-237 (2007). <https://doi.org/10.1038/sj.bjc.6603849>

17 Kawada, N. *et al.* Interferon gamma stimulates prostaglandin E2 production by mouse Kupffer cells. *Prostaglandins Leukot Essent Fatty Acids* **40**, 275-279 (1990). <https://doi.org/10.1016/0952-3278(90)90049-q>

18 Chawansuntati, K., Rattanathammethee, K. & Wipasa, J. Minireview: Insights into anti-interferon-γ autoantibodies. *Exp Biol Med (Maywood)* **246**, 790-795 (2021). <https://doi.org/10.1177/1535370220981579>

19 Shih, H. P. *et al.* Pathogenic autoantibodies to IFN-γ act through the impedance of receptor assembly and Fc-mediated response. *J Exp Med* **219** (2022). <https://doi.org/10.1084/jem.20212126>

20 Jauhar, S. *et al.* A leaky umbrella has little value: evidence clearly indicates the serotonin system is implicated in depression. *Mol Psychiatry* **28**, 3149-3152 (2023). <https://doi.org/10.1038/s41380-023-02095-y>

21 Basselin, M. *et al.* Imaging Elevated Brain Arachidonic Acid Signaling in Unanesthetized Serotonin Transporter (5-HTT)-Deficient Mice. *Neuropsychopharmacology* **34**, 1695-1709 (2009). <https://doi.org/10.1038/npp.2008.227>

22 Singh, A. K. *et al.* Prostaglandin-mediated inhibition of serotonin signaling controls the affective component of inflammatory pain. *J Clin Invest* **127**, 1370-1374 (2017). <https://doi.org/10.1172/jci90678>

23 Günther, J., Schulte, K., Wenzel, D., Malinowska, B. & Schlicker, E. Prostaglandins of the E series inhibit monoamine release via EP3 receptors: proof with the competitive EP3 receptor antagonist L-826,266. *Naunyn Schmiedebergs Arch Pharmacol* **381**, 21-31 (2010). <https://doi.org/10.1007/s00210-009-0478-9>

24 Schott, K. *et al.* Autoantibodies to serotonin in serum of patients with psychiatric disorders. *Psychiatry Research* **121**, 51-57 (2003). <https://doi.org/https://doi.org/10.1016/S0165-1781(03)00137-9>
