# Supplementary material for: Persistent prostaglandin E2 upregulation and hormonal multi-resistance: A hypothesis for long COVID
Source: Biochem Biophys Rep. 2026 Mar 10;46:102518. doi: 10.1016/j.bbrep.2026.102518 (PMC12994086; doi:10.1016/j.bbrep.2026.102518)
Supplement: Multimedia component 3 [file mmc3.docx]

Multimedia component 3. Four LC sub phenotypes and clinical consequences of a validated PGE2 hypothesis

**1. Hypothetical EP receptor distribution in four sub phenotypes of LC patients**

Zhang et al. investigated the incidence and reproducibility of specific Long Covid-symptoms in individuals who contracted COVID19 (in the pre-Omicron period) [^1^](#_ENREF_1). In a development cohort of 20.881 and a validation cohort of 13.724 patients, they identified over 137 Long Covid symptoms and conditions in four reproducible subphenotypes [^1^](#_ENREF_1).

- Type 1 “Cardiac and renal”: Compared to the other subphenotypes patients were older, mean age 65 yrs, predominantly men, with more severe illness and the highest hospitalization rate. They also had the highest burden of comorbidities, ao cardiac and circulatory problems and kidney problems
- Type 2 “Respiratory, sleep and anxiety”: consisted of more women, mean age 51 yrs.
- Type 3 “Musculoskeletal and nervous”: more women and higher baseline comorbidity of autoimmune and allergy conditions, mean age 57 yrs.
- Type 4 “Digestive and respiratory”, smallest cohort, mainly female patients, digestive system, urinary and respiratory conditions, lower burden of underlying conditions, mean age 54 yrs.

We considered Zhang’s data according to our ‘EP receptor up and down’ approach (Data-set 1.). We collected evidence that the symptoms of Zhangs “Type 1” can be correlated with “EP3 up”. The symptoms of the other three types can be linked to “EP3 down” combined with “EP1 up”, “EP2 up” and “EP4 up” respectively (see for references Data-set 2).
Of significance: EP1, EP2 and EP4 are expected to upregulate by infections while EP3 expression is expected to downregulate, looking at the reported effect of PGE2/COX-2 upregulation on EP receptor expressions (see main text, section 3.1).

**Data-set 1. Hypothetical EP receptor distribution in four sub phenotypes of LC patients**

| Zhangs 4 types of Long COVID patients | Characteristic LC subphenotypes according to Zhangs research  and terminology | Male/female ratio | Proposed distribution patterns of four EP expression based on our literature searches (see references in Data-set 1) compared with the presumed profiles of the other types and average EP expressions | |
| --- | --- | --- | --- | --- |
| **Type 1** | “Cardiac and renal” | Mostly male | EP3 up |  |
| **Type 2** | “Respiratory, sleep  and anxiety” | Mostly female | EP1 up | **EP3 down** |
| **Type 3** | “Musculoskeletal and nervous” | Mostly female | EP2 up | **EP3 down** |
| **Type 4** | “Digestive and respiratory” | Mostly female | EP4 up | **EP3 up/down*** |
| * EP4 expression (↑cAMP) up = EP3 capacity (↓cAMP) down, even when EP3 expression is up | | | | |

**Data-set 2. Four LC symptom clusters hypothetically associated with dominance of a distinct EP receptor**

| Type 1 “Cardiac and renal”  EP3 up  PGE2-EP3 activity contributes to high blood pressure [^2^](#_ENREF_2)^,^[^3^](#_ENREF_3) and cardiac [^4-8^](#_ENREF_4) and renal  [^9-12^](#_ENREF_9) problems | Type 4 “Digestive and respiratory”   EP4 up    PGE2-EP4 activity stimulates bile [^13^](#_ENREF_13) and reportedly contributes to gastrointestinal [^14-16^](#_ENREF_14) and airway [^17^](#_ENREF_17)^,^[^18^](#_ENREF_18) problems  – bile stimulates both [^19^](#_ENREF_19)^,^[^20^](#_ENREF_20) |
| --- | --- |
| Type 2 “Respiratory, sleep and anxiety”  EP1 up   PGE2-EP1 activity contributes to respiratory problems [^21-24^](#_ENREF_21) and mediates elevated anxiety [^25^](#_ENREF_25). PGE2 and COX-2 derived PGD2 through EP1 have opposing roles in sleep regulation [^26^](#_ENREF_26) | Type 3 “Musculoskeletal and nervous”   EP2 up    PGE2-EP2 activity stimulates oligodendrocyte apoptosis and demyelination, possibly contributing to musculoskeletal [^27-30^](#_ENREF_27)^,^[^31^](#_ENREF_31) and nervous [^32-35^](#_ENREF_32) problems. PGE2-EP2 stimulates aldosterone [^36^](#_ENREF_36) and aldosterone upregulation is associated with damages to skeletal muscle cells [^37^](#_ENREF_37)^,^[^38^](#_ENREF_38) |

These models might help finding the right treatment for more specific types of Long COVID patients.

**2. Potential strategies to modulate PGE2 and EP receptor activity**

In Data-set 3. below, we summarize several reported and hypothetical strategies to modulate PGE2 and EP receptor activity as potential theraputic targets for LC. We propose that PGE2 plays a principal role in LC and that EP receptor expression differs among patients, which may necessitate different therapeutic interventions. As mentioned in the main article regarding COX-2-lowering medications, a substantial decrease in PGE2 levels could upregulate EP3 receptors while downregulating EP1, EP2, and EP4 (see main text, section 3.1). Imbalances in EP expression could be reduced (especially in Category B - “EP3 down”) but also exacerbated (especially in Category A - “EP3 up”) (See Table 3 in section 7.5). Consequently, certain PGE2-lowering interventions may be beneficial for some patient subsets but ineffective - or even harmful - for others.
We propose that interventions which reduce PGE2 levels while markedly increasing blood pressure [^39^](#_ENREF_39) may be more beneficial for the “EP3 down” category, given that PGE2 stimulates BP primarily via EP3 signaling. In this context, a reduction in PGE2 activity would not be expected to increase BP unless EP3 capacity is significantly enhanced. Conversely, interventions that attenuate PGE2 signaling within the sympathetic nervous system (SNS) may be more appropriate for the “EP3 up” category, where they could help modulate excessive EP3-mediated signaling. For hypothetical EP3 up or EP3 down biomarkers, see main text, Table 3. in section 7.5.

**Data-set 3. Potential strategies to modulate PGE2 and EP receptor activity**

| **Hypothetical potential intervention:** | | **Hypothetical mechanisms of action:** | | |
| --- | --- | --- | --- | --- |
| Pharmaceuticals that could rebalance EP receptor availability  - not tested for Long COVID - | | | | |
| **Pharmacological  EP-antagonists/agonists** | | Agonism or antagonism of specific EP receptors may represent a potential strategy to adjust imbalanced physiological EP receptor signaling in patients with long COVID | | |
| **EP1** ANTAGONISTS  SC19220 [^40^](#_ENREF_40)  ONO-AE829 [^41^](#_ENREF_41)  AH6809 [^42^](#_ENREF_42) | **EP1** AGONISTS  Iloprost[^40^](#_ENREF_40)   17-phenyl-trinor-PGE(2) [^42^](#_ENREF_42)^,^[^43^](#_ENREF_43) | | **EP2** ANTAGONISTS  AH6809 [^44^](#_ENREF_44)   PF-04418948 [^45^](#_ENREF_45) | **EP2** AGONISTS  Butaprost[^40^](#_ENREF_40)   ONO-AE1-259 [^31^](#_ENREF_31) |
| **EP3** ANTAGONISTS  2-amino thiadiazole amiden [^46^](#_ENREF_46)  ONO-AE3-240 [^47^](#_ENREF_47)  AE5-599 [^48^](#_ENREF_48) AE3-240 [^48^](#_ENREF_48) | **EP3** AGONISTS  Sulprostone [^49^](#_ENREF_49) ONO-AE3-208 [^47^](#_ENREF_47)   BGC20-1531 [^50^](#_ENREF_50) ONO-AE-248 [^47^](#_ENREF_47)^,^[^51^](#_ENREF_51)^,^[^52^](#_ENREF_52) | | **EP4** ANTAGONISTS  Enprostil [^53^](#_ENREF_53)  ONO-NT012 [^54^](#_ENREF_54) | **EP4** AGONISTS  ONO-AE1-329 [^55^](#_ENREF_55)  and more  full agonists [^56^](#_ENREF_56) |
| Medication that reportedly decreases PGE2 acitvity - not tested for Long COVID - | | | | |

| **PGE1- [34] and PGE3-analogs** [^57^](#_ENREF_57) | These analogs can reduce the adverse influence of PGE2 by binding to the EP-receptors as rival ligands [^57^](#_ENREF_57).   - may provide benefits across the four sub-phenotypes |
| --- | --- |
| **15-PGDH-activators** [^58^](#_ENREF_58) | 15-PGDH degrades PGE2 (and some other prostaglandins) into its inactive metabolite. Increasing 15-PGDH could help to decrease PGE2 activity more selectively than with NSAIDs [^58^](#_ENREF_58).   - potentially safer combined with EP receptor agonism or inhibition |
| **mPGES-1 inhibitors**  Sulforaphane [^59^](#_ENREF_59)  - other mPGES-1 inhibitors  or suppressors [^60-62^](#_ENREF_60)  - Dual COX-LOX inhibitors [^63^](#_ENREF_63) (revi  **NSAIDs combined with EP receptor agonism or inhibition**  Example with EP4 antagonist ONO-AE3-208 [^64^](#_ENREF_64) | These medications target steps in the synthesis or degradation of PGE2, possibly in combination with EP-receptor (ant)agonists, to decrease side effects   - potentially safer combined with EP receptor agonism or inhibition |
| **Metformin**  Review on LC interventions [^65^](#_ENREF_65)  “Early findings suggest that metformin has the strongest clinical evidence, particularly from large phase 3 trials” | Metformin reduces PGE2 activity [^66^](#_ENREF_66)^,^ [^67^](#_ENREF_67)^,^[^68^](#_ENREF_68)   - potentially more beneficial “for EP3 up” - potentially contra-indicated for “EP3 down”   see section 2.1 below |
| Pharmalogical agent that reportedly can increase EP3 expression  - not tested for Long COVID - | |
| **Demethylating DNA with 5-aza-dC** to increase expression of EP3 receptors[^69^](#_ENREF_69) | This compound has been shown to increase/restore the expression of EP3-receptors by demethylation [^69^](#_ENREF_69)   - Contra-indicated for “EP3 up” |
| Psychopharmacological agents that elevate monoamine levels  through shared subcellular signaling pathways monoamines may reduce PGE2 activity  (but they could increase it aswell, depending on the dose and other factors). (see main text, Sections 5.1.4, 9.2 and 10. on monoamine-PGE2 interactions)  - tested for Long COVID, partially succesful - | |
| **Methylphenidate**  Two LC case series [^70^](#_ENREF_70)^,^[^71^](#_ENREF_71) | Methylphenidate increases DA levels [^72^](#_ENREF_72)  (See main text, sections 5.1.4) |
| **Bupropion**  LC case series [^73^](#_ENREF_73) | Bupropion is a DA and noradrenalin reuptake inhibitor, increasing their levels [^73^](#_ENREF_73).  (See main text, sections 5.1.4) |
| **SSRI’s** [^74^](#_ENREF_74)^,^[^75^](#_ENREF_75)  “After a year, chronic fatigue, dysautonomia and neurological and neuropsychiatric complaints predominate. In this study, 95 PCS patients were treated with selective serotonin reuptake inhibitors” [^61^](#_ENREF_61) | SSRI’s raise serotonin levels   “This study used an exploratory questionnaire and found that *two-thirds* of patients had a reasonably good to strong response on SSRIs, over a quarter of patients had moderate response, while 10% reported no response”  (See main text, sections 5.1.4) |
| - not tested for long COVID - | |
| **Melatonin**  We found no melatonin trial for long COVID  “On the protective effect of melatonin in severe-*COVID-19* patients. The protocol consisted of standard therapy with oxygen intubation, remdesivir, levofloxacin, dexamethasone, and enoxaparin. Half of the patients additionally received 10 mg of melatonin. Interestingly, the mortality rate was 17.1% in the conventional therapy group and 1.2% in melatonin group; thus, overall, the mortality was reduced by 93% in patients treated with melatonin”. [^76^](#_ENREF_76) | Melatonin is reported to decrease PGE2 activity by modulating cAMP [^77^](#_ENREF_77)^,^[^78^](#_ENREF_78)   - potentially more beneficial “for EP3 up” - potentially contra-indicated for “EP3 down”   see section 2.2 below on modulating cAMP |
| **Immunoadsorption**  Preliminary studies on the treatment of ME/CFS patients [^79^](#_ENREF_79)  “Preliminary studies on the treatment of ME/CFS patients with immunoadsorption (IA), an apheresis that removes antibodies from plasma, suggest clinical improvement” | Removes IgG type autoantibodies  Review article “Chronic inflammation in Long COVID relationship to autoimmune diseases” [^80^](#_ENREF_80)  “We intervene in the treatment of the disease with probiotics, immunoglobulins, the RECOVER clinical trial model, and immunomodulatory drugs. The aim is to enhance understanding of the pathophysiological mechanism of Long COVID and to provide a reference for the immunotherapy of patients.”   - potentially beneficial for patients with EP2 up and/or EP4 up (“EP3 down”) |

**2.1. Modulating EP receptor balance and PGE2 activity by modulating extracellular Ca2+ levels and AMP kinase?**

Metformin activates adenosine monophosphate kinase (AMPK) [^81^](#_ENREF_81), modulates Ca2+ levels [^82^](#_ENREF_82) and can inhibit PGE2 activity via AMPK [^67^](#_ENREF_67)^,^[^68^](#_ENREF_68). EP1 and EP3 are partially Ca2+-coupled, while EP2 and EP4 signal through cAMP (cyclic adenosine monophosphate) [^83^](#_ENREF_83). Activating AMPK could increase EP2 and EP4 signaling through cAMP [^83^](#_ENREF_83). Thus, drugs that influence AMPK and/or Ca2+ may modulate EP receptor signaling and PGE2 activity, warranting further investigation.

**2.2.** **Could lidocaine function as an EP1 antagonist, decreasing Ca2+ levels?**

EP1 and lidocaine exert many opposite functions, illlustrated by a few examples below.

| EP1 | Lidocaine |
| --- | --- |
| 1. stimulates Ca2+ [^84^](#_ENREF_84) | 1. Lidocaine inhibits increase of Ca2+ [^85^](#_ENREF_85) |
| 2. stimulates pain [^86^](#_ENREF_86)^,^[^87^](#_ENREF_87) | 2. decreases pain [^88^](#_ENREF_88)^,^[^89^](#_ENREF_89) |
| 3. stimulates uterus contractions [^90^](#_ENREF_90) | 3. decreases uterus contractions [^91^](#_ENREF_91) |
| 4. stimulates CKD [^92^](#_ENREF_92) | 4. decreases CKD [^93^](#_ENREF_93) |
| 5. stimulates miosis [^94^](#_ENREF_94) | 5. stimulates midriasis [^95^](#_ENREF_95) |

Lidocaine seems to function as an EP1 antagonist. It may hypothetically inhibit PGE2 signaling via EP1 through interaction with the Gq subunit. (20). The following data support this hypothesis.

- EP1 has a Gq sub-unit (20)
- Lidocaine reportedly binds Gq subunits [^89^](#_ENREF_89)
- EP1 stimulates Ca2+ [^84^](#_ENREF_84)
- Lidocaine inhibits increase of Ca2+ [^85^](#_ENREF_85)
- Lidocaine antagonizes P2X7 activity [^96^](#_ENREF_96)
- P2X7 elevates intracellular Ca²⁺ [^97^](#_ENREF_97)
- P2X7 stimulates COX-2/ PGE2 activity [^98^](#_ENREF_98)
- Soluble P2X7 receptor is elevated in the plasma of COVID-19 Patients [^99^](#_ENREF_99)
- Chronic Lidocaïne administration reportedly decreases COX-2 /PGE2 activity [^100^](#_ENREF_100)

In the main text, Section 5.1.2, we discuss the possibility that PGE2 may stimulate detrimental phosphorylation processes via EP1 signaling. We hypothesize that lidocaine could interfere with these processes and thereby potentially alleviate long COVID symptoms, particularly in the cognitive domain. A lack of response to chronic lidocaine treatment might be attributable, at least in part, to insufficient EP1 receptor expression.

References

1 Zhang, H. *et al.* Data-driven identification of post-acute SARS-CoV-2 infection subphenotypes. *Nature Medicine* **29**, 226-235 (2023). <https://doi.org/10.1038/s41591-022-02116-3>

2 Bryson, T. *et al.* The Deleterious Role of the Prostaglandin E 2 EP3 Receptor in Angiotensin II Hypertension. *American Journal of Physiology-Heart and Circulatory Physiology* **318** (2020). <https://doi.org/10.1152/ajpheart.00538.2019>

3 Wu, J. *et al.* EP3 (E-Prostanoid 3) Receptor Mediates Impaired Vasodilation in a Mouse Model of Salt-Sensitive Hypertension. *Hypertension* **77**, 1399-1411 (2021). <https://doi.org/doi:10.1161/HYPERTENSIONAHA.120.16518>

4 Bryson, T. D. *et al.* Targeted Gene Deletion or Antagonism of the Prostaglandin E2 EP3 Receptor Protects Against Cardiac Injury Postmyocardial Infarction. *Circulation: Heart Failure* **17**, e011089 (2024). <https://doi.org/doi:10.1161/CIRCHEARTFAILURE.123.011089>

5 Schaid, M. D., Wisinski, J. A. & Kimple, M. E. The EP3 Receptor/G(z) Signaling Axis as a Therapeutic Target for Diabetes and Cardiovascular Disease. *Aaps j* **19**, 1276-1283 (2017). <https://doi.org/10.1208/s12248-017-0097-1>

6 Gu, X. *et al.* Prostaglandin E2 Reduces Cardiac Contractility via EP3 Receptor. *Circ Heart Fail* **9** (2016). <https://doi.org/10.1161/CIRCHEARTFAILURE.116.003291>

7 Meyer-Kirchrath, J. *et al.* Overexpression of prostaglandin EP3 receptors activates calcineurin and promotes hypertrophy in the murine heart. *Cardiovasc Res* **81**, 310-318 (2009). <https://doi.org/10.1093/cvr/cvn312>

8 Mawhin, M. A., Tilly, P. & Fabre, J. E. The receptor EP3 to PGE2: A rational target to prevent atherothrombosis without inducing bleeding. *Prostaglandins Other Lipid Mediat* **121**, 4-16 (2015). <https://doi.org/10.1016/j.prostaglandins.2015.10.001>

9 Jing, L., Wen, Z. & Bin, L. The role of E-type prostaglandin receptor EP3 in acute renal injury induced by ischemia-reperfusion. *Journal of Molecular and Cellular Cardiology* **140**, 56 (2020). <https://doi.org/https://doi.org/10.1016/j.yjmcc.2019.11.135>

10 Leng, J. *et al.* E-prostanoid 3 receptor deficiency on myeloid cells protects against ischemic acute kidney injury via breaking the auto-amplification loop of necroinflammation. *Kidney International* **103**, 100-114 (2023). <https://doi.org/https://doi.org/10.1016/j.kint.2022.08.019>

11 Hassouneh, R. *et al.* PGE2 receptor EP3 inhibits water reabsorption and contributes to polyuria and kidney injury in a streptozotocin-induced mouse model of diabetes. *Diabetologia* **59**, 1318-1328 (2016). <https://doi.org/10.1007/s00125-016-3916-5>

12 Vio, C. P., Quiroz-Munoz, M., Cuevas, C. A., Cespedes, C. & Ferreri, N. R. Prostaglandin E2 EP3 receptor regulates cyclooxygenase-2 expression in the kidney. *Am J Physiol Renal Physiol* **303**, F449-457 (2012). <https://doi.org/10.1152/ajprenal.00634.2011>

13 Ying, F. *et al.* EP4 emerges as a novel regulator of bile acid synthesis and its activation protects against hypercholesterolemia. *Biochimica et Biophysica Acta (BBA) - Molecular and Cell Biology of Lipids* **1863**, 1029-1040 (2018). <https://doi.org/https://doi.org/10.1016/j.bbalip.2018.06.003>

14 Dey, I., Giembycz, M. A. & Chadee, K. Prostaglandin E(2) couples through EP(4) prostanoid receptors to induce IL-8 production in human colonic epithelial cell lines. *Br J Pharmacol* **156**, 475-485 (2009). <https://doi.org/10.1111/j.1476-5381.2008.00056.x>

15 Crittenden, S. *et al.* *Prostaglandin E2 promotes intestinal inflammation via inhibiting microbiota-dependent regulatory T cells*. (2020).

16 Maseda, D. *et al.* mPGES-1-Mediated Production of PGE2 and EP4 Receptor Sensing Regulate T Cell Colonic Inflammation. *Frontiers in Immunology* **Volume 9 - 2018** (2018). <https://doi.org/10.3389/fimmu.2018.02954>

17 Akaba, T. *et al.* Activating prostaglandin E2 receptor subtype EP4 increases secreted mucin from airway goblet cells. *Pulmonary Pharmacology & Therapeutics* **48**, 117-123 (2018). <https://doi.org/https://doi.org/10.1016/j.pupt.2017.11.001>

18 Aso, H. *et al.* Prostaglandin E2 enhances interleukin-8 production via EP4 receptor in human pulmonary microvascular endothelial cells. *American Journal of Physiology-Lung Cellular and Molecular Physiology* **302**, L266-L273 (2011). <https://doi.org/10.1152/ajplung.00248.2011>

19 Reen, F. J. *et al.* Bile signalling promotes chronic respiratory infections and antibiotic tolerance. *Sci Rep* **6**, 29768 (2016). <https://doi.org/10.1038/srep29768>

20 Flynn, S. *et al.* Bile Acid Signal Molecules Associate Temporally with Respiratory Inflammation and Microbiome Signatures in Clinically Stable Cystic Fibrosis Patients. *Microorganisms* **8** (2020). <https://doi.org/10.3390/microorganisms8111741>

21 McGraw, D. W. *et al.* Airway smooth muscle prostaglandin-EP1 receptors directly modulate beta2-adrenergic receptors within a unique heterodimeric complex. *J Clin Invest* **116**, 1400-1409 (2006). <https://doi.org/10.1172/jci25840>

22 Okazaki, A. *et al.* Role of prostaglandin E2 in bronchoconstriction-triggered cough response in guinea pigs. *Pulmonary Pharmacology & Therapeutics* **48**, 62-70 (2018). <https://doi.org/https://doi.org/10.1016/j.pupt.2017.09.003>

23 Tilley, S. L. *et al.* Receptors and pathways mediating the effects of prostaglandin E2 on airway tone. *American Journal of Physiology-Lung Cellular and Molecular Physiology* **284**, L599-L606 (2003). <https://doi.org/10.1152/ajplung.00324.2002>

24 Okazaki, A. *et al.* Role of prostaglandin E(2) in bronchoconstriction-triggered cough response in guinea pigs. *Pulm Pharmacol Ther* **48**, 62-70 (2018). <https://doi.org/10.1016/j.pupt.2017.09.003>

25 Kitaoka, S. & Furuyashiki, T. [Roles of inflammation-related molecules in emotional changes induced by repeated stress]. *Nihon Shinkei Seishin Yakurigaku Zasshi* **34**, 109-115 (2014).

26 Yoshida, Y. *et al.* Prostaglandin E (EP) receptor subtypes and sleep: promotion by EP4 and inhibition by EP1/EP2. *Neuroreport* **11**, 2127-2131 (2000). <https://doi.org/10.1097/00001756-200007140-00014>

27 Gao, X. *et al.* Targeting EP2 Receptor Improves Muscle and Bone Health in Dystrophin−/−/Utrophin−/− Double-Knockout Mice. *Cells* **14**, 116 (2025).

28 Nango, H. *et al.* Update on the pathological roles of prostaglandin E(2) in neurodegeneration in amyotrophic lateral sclerosis. *Transl Neurodegener* **12**, 32 (2023). <https://doi.org/10.1186/s40035-023-00366-w>

29 Wang, P., Zhu, F., Lee, N. H. & Konstantopoulos, K. Shear-induced interleukin-6 synthesis in chondrocytes: roles of E prostanoid (EP) 2 and EP3 in cAMP/protein kinase A- and PI3-K/Akt-dependent NF-kappaB activation. *J Biol Chem* **285**, 24793-24804 (2010). <https://doi.org/10.1074/jbc.M110.110320>

30 Sato, T. *et al.* Prostaglandin EP2 receptor signalling inhibits the expression of matrix metalloproteinase 13 in human osteoarthritic chondrocytes. *Ann Rheum Dis* **70**, 221-226 (2011). <https://doi.org/10.1136/ard.2009.118620>

31 Ota, H. *et al.* EP2 receptor plays pivotal roles in generating mechanical hyperalgesia after lengthening contractions. *Scand J Med Sci Sports* **28**, 826-833 (2018). <https://doi.org/10.1111/sms.12954>

32 Lushington, R., Camilli, S., Pascual, F., Lockey, R. F. & Kolliputi, N. EP2 inhibition restores myeloid metabolism and reverses cognitive decline. *Journal of Allergy and Clinical Immunology: Global* **2**, 100082 (2023). <https://doi.org/https://doi.org/10.1016/j.jacig.2023.100082>

33 Kang, X. *et al.* Cyclooxygenase-2 contributes to oxidopamine-mediated neuronal inflammation and injury via the prostaglandin E2 receptor EP2 subtype. *Scientific Reports* **7**, 9459 (2017). <https://doi.org/10.1038/s41598-017-09528-z>

34 Minhas, P. S. *et al.* Restoring metabolism of myeloid cells reverses cognitive decline in ageing. *Nature* **590**, 122-128 (2021). <https://doi.org/10.1038/s41586-020-03160-0>

35 Palumbo, S., Toscano, C. D., Parente, L., Weigert, R. & Bosetti, F. The cyclooxygenase-2 pathway via the PGE₂ EP2 receptor contributes to oligodendrocytes apoptosis in cuprizone-induced demyelination. *J Neurochem* **121**, 418-427 (2012). <https://doi.org/10.1111/j.1471-4159.2011.07363.x>

36 Csukas, S., Hanke, C. J., Rewolinski, D. & Campbell, W. B. Prostaglandin E2-induced aldosterone release is mediated by an EP2 receptor. *Hypertension* **31**, 575-581 (1998). <https://doi.org/10.1161/01.hyp.31.2.575>

37 Kang, B. *et al.* Elevated Myoglobin in Patients With Primary Aldosteronism: A Cross-Sectional Study. *Front Endocrinol (Lausanne)* **13**, 799174 (2022). <https://doi.org/10.3389/fendo.2022.799174>

38 Palmer, B. F. & Clegg, D. J. Extrarenal Effects of Aldosterone on Potassium Homeostasis. *Kidney360* **3**, 561-568 (2022). <https://doi.org/10.34067/kid.0006762021>

39 Shungin, D. *et al.* New genetic loci link adipose and insulin biology to body fat distribution. *Nature* **518**, 187-196 (2015). <https://doi.org/10.1038/nature14132>

40 Botella, A., Delvaux, M., Fioramonti, J., Frexinos, J. & Bueno, L. Stimulatory (EP1 and EP3) and inhibitory (EP2) prostaglandin E2 receptors in isolated ileal smooth muscle cells. *Eur J Pharmacol* **237**, 131-137 (1993). <https://doi.org/10.1016/0014-2999(93)90102-n>

41 Takeuchi, K., Araki, H., Umeda, M., Komoike, Y. & Suzuki, K. Adaptive gastric cytoprotection is mediated by prostaglandin EP1 receptors: a study using rats and knockout mice. *J Pharmacol Exp Ther* **297**, 1160-1165 (2001).

42 Rutkai, I. *et al.* Activation of prostaglandin E2 EP1 receptor increases arteriolar tone and blood pressure in mice with type 2 diabetes. *Cardiovasc Res* **83**, 148-154 (2009). <https://doi.org/10.1093/cvr/cvp098>

43 Araki, H. *et al.* The roles of prostaglandin E receptor subtypes in the cytoprotective action of prostaglandin E2 in rat stomach. *Aliment Pharmacol Ther* **14 Suppl 1**, 116-124 (2000). <https://doi.org/10.1046/j.1365-2036.2000.014s1116.x>

44 Li, P. *et al.* AH6809 decreases production of inflammatory mediators by PGE(2) - EP2 - cAMP signaling pathway in an experimentally induced pure cerebral concussion in rats. *Brain Res* **1698**, 11-28 (2018). <https://doi.org/10.1016/j.brainres.2018.05.030>

45 Säfholm, J. *et al.* Prostaglandin E2 inhibits mast cell-dependent bronchoconstriction in human small airways through the E prostanoid subtype 2 receptor. *J Allergy Clin Immunol* **136**, 1232-1239.e1231 (2015). <https://doi.org/10.1016/j.jaci.2015.04.002>

46 Hilfiker, M. *et al.* Discovery of novel aminothiadiazole amides as selective EP3 receptor antagonists. *Bioorganic & medicinal chemistry letters* **19**, 4292-4295 (2009). <https://doi.org/10.1016/j.bmcl.2009.05.074>

47 Nagano, T. *et al.* Prostaglandin E(2) increases the expression of cyclooxygenase-2 in cultured rat microglia. *J Neuroimmunol* **361**, 577724 (2021). <https://doi.org/10.1016/j.jneuroim.2021.577724>

48 Schober, L. J. *et al.* The role of PGE2 in human atherosclerotic plaque on platelet EP3 and EP4 receptor activation and platelet function in whole blood. *Journal of Thrombosis and Thrombolysis* **32**, 158-166 (2011). <https://doi.org/10.1007/s11239-011-0577-6>

49 Nazabal, A., Mendiguren, A. & Pineda, J. Inhibition of rat locus coeruleus neurons by prostaglandin E2 EP3 receptors: pharmacological characterization ex vivo. *Frontiers in Pharmacology* **14** (2023). <https://doi.org/10.3389/fphar.2023.1290605>

50 Antonova, M. *et al.* The pharmacological effect of BGC20-1531, a novel prostanoid EP4 receptor antagonist, in the prostaglandin E2 human model of headache. *J Headache Pain* **12**, 551-559 (2011). <https://doi.org/10.1007/s10194-011-0358-9>

51 Mori, A. *et al.* Effects of specific prostanoid EP receptor agonists on cell proliferation and intracellular Ca(2+) concentrations in human airway smooth muscle cells. *Eur J Pharmacol* **659**, 72-78 (2011). <https://doi.org/10.1016/j.ejphar.2011.03.001>

52 Minami, T. *et al.* Characterization of EP receptor subtypes responsible for prostaglandin E2-induced pain responses by use of EP1 and EP3 receptor knockout mice. *Br J Pharmacol* **133**, 438-444 (2001). <https://doi.org/10.1038/sj.bjp.0704092>

53 Takeuchi, K., Yagi, K., Kato, S. & Ukawa, H. Roles of prostaglandin E-receptor subtypes in gastric and duodenal bicarbonate secretion in rats. *Gastroenterology* **113**, 1553-1559 (1997). <https://doi.org/10.1053/gast.1997.v113.pm9352857>

54 Hase, S., Yokota, A., Nakagiri, A. & Takeuchi, K. Prostaglandin E2 aggravates gastric mucosal injury induced by histamine in rats through EP1 receptors. *Life Sciences* **74**, 629-641 (2003). <https://doi.org/https://doi.org/10.1016/j.lfs.2003.07.010>

55 Ariumi, H. *et al.* Roles of the central prostaglandin EP3 receptors in cardiovascular regulation in rats. *Neurosci Lett* **324**, 61-64 (2002). <https://doi.org/10.1016/s0304-3940(02)00174-x>

56 Wilson, R. J. *et al.* Functional pharmacology of human prostanoid EP2 and EP4 receptors. *Eur J Pharmacol* **501**, 49-58 (2004). <https://doi.org/10.1016/j.ejphar.2004.08.025>

57 Robertson, R. P. The COX-2/PGE(2)/EP3/G(i/o)/cAMP/GSIS Pathway in the Islet: The Beat Goes On. *Diabetes* **66**, 1464-1466 (2017). <https://doi.org/10.2337/dbi17-0017>

58 Sun, C. C. *et al.* Recent advances in studies of 15-PGDH as a key enzyme for the degradation of prostaglandins. *Int Immunopharmacol* **101**, 108176 (2021). <https://doi.org/10.1016/j.intimp.2021.108176>

59 Zhou, J., Joplin, D. G., Cross, J. V. & Templeton, D. J. Sulforaphane Inhibits Prostaglandin E2 Synthesis by Suppressing Microsomal Prostaglandin E Synthase 1. *PLOS ONE* **7**, e49744 (2012). <https://doi.org/10.1371/journal.pone.0049744>

60 Park, J. Y., Pillinger, M. H. & Abramson, S. B. Prostaglandin E2 synthesis and secretion: The role of PGE2 synthases. *Clinical Immunology* **119**, 229-240 (2006). <https://doi.org/https://doi.org/10.1016/j.clim.2006.01.016>

61 Khan, H. *et al.* Plant-derived mPGES-1 inhibitors or suppressors: A new emerging trend in the search for small molecules to combat inflammation. *European Journal of Medicinal Chemistry* **153**, 2-28 (2018). <https://doi.org/https://doi.org/10.1016/j.ejmech.2017.12.059>

62 Ikeda-Matsuo, Y. *et al.* Microsomal prostaglandin E synthase-1 is a critical factor in dopaminergic neurodegeneration in Parkinson's disease. *Neurobiology of Disease* **124**, 81-92 (2019). <https://doi.org/https://doi.org/10.1016/j.nbd.2018.11.004>

63 Mukhopadhyay, N., Shukla, A., Makhal, P. N. & Kaki, V. R. Natural product-driven dual COX-LOX inhibitors: Overview of recent studies on the development of novel anti-inflammatory agents. *Heliyon* **9**, e14569 (2023). <https://doi.org/10.1016/j.heliyon.2023.e14569>

64 Pavlovic, S. *et al.* Targeting prostaglandin E2 receptors as an alternative strategy to block cyclooxygenase-2-dependent extracellular matrix-induced matrix metalloproteinase-9 expression by macrophages. *J Biol Chem* **281**, 3321-3328 (2006). <https://doi.org/10.1074/jbc.M506846200>

65 Livieratos, A., Gogos, C. & Akinosoglou, K. Beyond Antivirals: Alternative Therapies for Long COVID. *Viruses* **16** (2024). <https://doi.org/10.3390/v16111795>

66 Han, Y. *et al.* Metformin decreases LPS-induced inflammatory response in rabbit annulus fibrosus stem/progenitor cells by blocking HMGB1 release. *Aging (Albany NY)* **11**, 10252-10265 (2019). <https://doi.org/10.18632/aging.102453>

67 Zhou, Y. *et al.* Metformin Suppresses Prostaglandin E2-Induced Cytochrome P450 Aromatase Gene Expression and Activity via Stimulation of AMP-Activated Protein Kinase in Human Endometriotic Stromal Cells. *Reprod Sci* **22**, 1162-1170 (2015). <https://doi.org/10.1177/1933719115590664>

68 Kalariya, N. M., Shoeb, M., Ansari, N. H., Srivastava, S. K. & Ramana, K. V. Antidiabetic drug metformin suppresses endotoxin-induced uveitis in rats. *Invest Ophthalmol Vis Sci* **53**, 3431-3440 (2012). <https://doi.org/10.1167/iovs.12-9432>

69 Fukushima, K. & Fujino, H. Identification and Characterization of Human Colorectal Cancer Cluster Predominantly Expressing EP3 Prostanoid Receptor Subtype. *Biological and Pharmaceutical Bulletin* **45**, 698-702 (2022). <https://doi.org/10.1248/bpb.b22-00104>

70 Clark, P. *et al.* Methylphenidate for the Treatment of Post-COVID Cognitive Dysfunction (Brain Fog). *J Med Cases* **15**, 195-200 (2024). <https://doi.org/10.14740/jmc4254>

71 Morelli-Zaher, C. *et al.* Post-COVID central hypersomnia, a treatable trait in long COVID: 4 case reports. *Front Neurol* **15**, 1349486 (2024). <https://doi.org/10.3389/fneur.2024.1349486>

72 Gottlieb, S. Methylphenidate works by increasing dopamine levels. *Bmj* **322**, 259 (2001). <https://doi.org/10.1136/bmj.322.7281.259>

73 Reinfeld, S. Can bupropion treat COVID-19–induced brain fog? A case series. *International Clinical Psychopharmacology* **38**, 189-191 (2023). <https://doi.org/10.1097/yic.0000000000000436>

74 Rus, C. P., de Vries, B. E. K., de Vries, I. E. J., Nutma, I. & Kooij, J. J. S. Treatment of 95 post-Covid patients with SSRIs. *Scientific Reports* **13**, 18599 (2023). <https://doi.org/10.1038/s41598-023-45072-9>

75 Seo, Y. B. *et al.* Therapeutic options for the treatment of post-acute sequelae of COVID-19: a scoping review. *BMC Infect Dis* **25**, 731 (2025). <https://doi.org/10.1186/s12879-025-11131-x>

76 Catalano, A. *et al.* Are Nutraceuticals Effective in COVID-19 and Post-COVID Prevention and Treatment? *Foods* **11** (2022). <https://doi.org/10.3390/foods11182884>

77 Carrillo-Vico, A., García-Mauriño, S., Calvo, J. R. & Guerrero, J. M. Melatonin counteracts the inhibitory effect of PGE2 on IL-2 production in human lymphocytes via its mt1 membrane receptor. *The FASEB Journal* **17**, 755-757 (2003). <https://doi.org/https://doi.org/10.1096/fj.02-0501fje>

78 Tse, L. H., Cheung, S. T., Lee, S. & Wong, Y. H. Real-Time Determination of Intracellular cAMP Reveals Functional Coupling of G(s) Protein to the Melatonin MT(1) Receptor. *Int J Mol Sci* **25** (2024). <https://doi.org/10.3390/ijms25052919>

79 Preßler, H. *et al.* IA-PACS-CFS: a double-blinded, randomized, sham-controlled, exploratory trial of immunoadsorption in patients with chronic fatigue syndrome (CFS) including patients with post-acute COVID-19 CFS (PACS-CFS). *Trials* **25**, 172 (2024). <https://doi.org/10.1186/s13063-024-07982-5>

80 Chen, K. *et al.* Chronic inflammation in Long COVID relationship to autoimmune diseases. *Autoimmun Rev* **24**, 103882 (2025). <https://doi.org/10.1016/j.autrev.2025.103882>

81 Kisfalvi, K., Eibl, G., Sinnett-Smith, J. & Rozengurt, E. Metformin disrupts crosstalk between G protein-coupled receptor and insulin receptor signaling systems and inhibits pancreatic cancer growth. *Cancer Res* **69**, 6539-6545 (2009). <https://doi.org/10.1158/0008-5472.Can-09-0418>

82 Nascimento Da Conceicao, V. *et al.* Metformin-induced activation of Ca(2+) signaling prevents immune infiltration/pathology in Sjogren's syndrome-prone mouse models. *J Transl Autoimmun* **7**, 100210 (2023). <https://doi.org/10.1016/j.jtauto.2023.100210>

83 Biringer, R. G. A Review of Prostanoid Receptors: Expression, Characterization, Regulation, and Mechanism of Action. *J Cell Commun Signal* **15**, 155-184 (2021). <https://doi.org/10.1007/s12079-020-00585-0>

84 Ma, Y. *et al.* Calcium Signal Pathway is Involved in Prostaglandin E2 Induced Cardiac Fibrosis in Cardiac Fibroblasts. *J Pharm Pharm Sci* **21**, 326-339 (2018). <https://doi.org/10.18433/jpps29322>

85 Kai, T. *et al.* Effects of lidocaine on intracellular Ca2+ and tension in airway smooth muscle. *Anesthesiology* **78**, 954-965 (1993). <https://doi.org/10.1097/00000542-199305000-00021>

86 Moriyama, T. *et al.* Sensitization of TRPV1 by EP1 and IP reveals peripheral nociceptive mechanism of prostaglandins. *Mol Pain* **1**, 3 (2005). <https://doi.org/10.1186/1744-8069-1-3>

87 Stock, J. L. *et al.* The prostaglandin E2 EP1 receptor mediates pain perception and regulates blood pressure. *J Clin Invest* **107**, 325-331 (2001). <https://doi.org/10.1172/jci6749>

88 Yang, X., Wei, X., Mu, Y., Li, Q. & Liu, J. A review of the mechanism of the central analgesic effect of lidocaine. *Medicine (Baltimore)* **99**, e19898 (2020). <https://doi.org/10.1097/md.0000000000019898>

89 Hollmann, Markus W., McIntire, William E., Garrison, James C. & Durieux, Marcel E. Inhibition of Mammalian Gq Protein Function by Local Anesthetics. *Anesthesiology* **97**, 1451-1457 (2002). <https://doi.org/10.1097/00000542-200212000-00017>

90 Spaziani, E. P., Tsibris, J. C., Hunt, L. T., Benoit, R. R. & O'Brien, W. F. The effect of interleukin-1 beta and interleukin-4 on the expression of prostaglandin receptors EP1 and EP3 in amnion WISH cells. *Am J Reprod Immunol* **38**, 279-285 (1997). <https://doi.org/10.1111/j.1600-0897.1997.tb00515.x>

91 Weinschenk, F. *et al.* Uterine contractility changes in a perfused swine uterus model induced by local anesthetics procaine, lidocaine, and ropivacaine. *PLoS One* **13**, e0206053 (2018). <https://doi.org/10.1371/journal.pone.0206053>

92 Kresse, J. C. *et al.* EP(1) receptor antagonism mitigates early and late stage renal fibrosis. *Acta Physiol (Oxf)* **234**, e13780 (2022). <https://doi.org/10.1111/apha.13780>

93 Xu, X. *et al.* Epidural block with lidocaine ameliorates kidney function deterioration and fibrosis of chronic kidney disease in rats. *Am J Med Sci* **368**, 660-667 (2024). <https://doi.org/10.1016/j.amjms.2024.08.020>

94 Mukhopadhyay, P., Bian, L., Yin, H., Bhattacherjee, P. & Paterson, C. A. Localization of EP1 and FP Receptors in Human Ocular Tissues by In Situ Hybridization. *Investigative Ophthalmology & Visual Science* **42**, 424-428 (2001).

95 Nikeghbali, A., Falavarjani, K. G. & Kheirkhah, A. Pupil dilation with intracameral lidocaine during phacoemulsification: Benefits for the patient and surgeon. *Indian J Ophthalmol* **56**, 63-64 (2008). <https://doi.org/10.4103/0301-4738.37598>

96 Okura, D. *et al.* Lidocaine preferentially inhibits the function of purinergic P2X7 receptors expressed in Xenopus oocytes. *Anesth Analg* **120**, 597-605 (2015). <https://doi.org/10.1213/ane.0000000000000585>

97 Samways, D. S., Li, Z. & Egan, T. M. Principles and properties of ion flow in P2X receptors. *Front Cell Neurosci* **8**, 6 (2014). <https://doi.org/10.3389/fncel.2014.00006>

98 Barberà-Cremades, M. *et al.* P2X7 receptor-stimulation causes fever via PGE2 and IL-1β release. *The FASEB Journal* **26**, 2951-2962 (2012). <https://doi.org/https://doi.org/10.1096/fj.12-205765>

99 García-Villalba, J. *et al.* Soluble P2X7 Receptor Is Elevated in the Plasma of COVID-19 Patients and Correlates With Disease Severity. *Frontiers in Immunology* **Volume 13 - 2022** (2022). <https://doi.org/10.3389/fimmu.2022.894470>

100 Cook, V. L. *et al.* Anti-inflammatory effects of intravenously administered lidocaine hydrochloride on ischemia-injured jejunum in horses. *Am J Vet Res* **70**, 1259-1268 (2009). <https://doi.org/10.2460/ajvr.70.10.1259>
