# Supplementary material for: Persistent prostaglandin E2 upregulation and hormonal multi-resistance: A hypothesis for long COVID
Source: Biochem Biophys Rep. 2026 Mar 10;46:102518. doi: 10.1016/j.bbrep.2026.102518 (PMC12994086; doi:10.1016/j.bbrep.2026.102518)
Supplement: Multimedia component 4 [file mmc4.docx]

Multimedia component 4. “The Intricate Relations between PGE2 and Dopamine and their possible revelance for long COVID”

**1. Dopamine and PGE2: are they modulators of each other's activity?**

- PGE2 is a major mediator of inflammation [1]
   DA has anti-inflammatory effects [2]
- Dopamine release can be inhibited by PGE2 through the prostaglandin EP3-receptor [3]
- PGE2 mediates systemic sickness responses to respiratory virus infection through its receptor EP3 [4]. Research shows that malaise and aversion could be caused in humans by upregulated levels of PGE2 because it attenuates DA activity [5]

*(We propose that this antagonism of DA-activity by PGE2 serves the purpose of sparing energy as long as there is physical or mental stress to deal with).*

- PGE2 is involved in bronchoconstriction through EP3 [6,7]
   DA (inhaled or infused) decreases histamine-induced bronchoconstriction in normal and asthmatic subjects [8]
- PGE2-receptor EP1 renders dopaminergic neurons selectively vulnerable to direct PGE2 neurotoxicity [9]
- Cyclooxygenase (COX2) is the enzyme that is responsible for biosynthesis of PGE2. Increased COX-2 is reported to cause oxidative protein modification and α-synuclein accumulation in dopaminergic cells [10], as seen in Parkinson’s disease [11-13]
- DA production and activity can be downregulated by PGE2 through EP1 [14,15]. COX-2/PGE2 can be downregulated through EP1 by one or more unknown ligands [16,17]. Lack of EP1 can lead to a hyperdopaminergic state [18]
- EP1 can form a dimer with D1 [19]. PGE2 can amplify both D1- and D2-receptor signaling through EP1. Dopamine receptor D2 agonists are potent in enhancing the release of arachidonic acid (AA) [20-24]; AA is the first building block needed to synthesize PGE2 [25]
- Aripiprazole, shown possibly effective against Long Covid symptoms [26], raises DA levels in the medial prefrontal cortex and the hippocampus [27]. Aripiprazole has antipsychotic effects, “dependent on its low intrinsic activity at dopamine D2 receptors”, presumably “because it does not overstimulate PGE2 formation” [28]

**2. A hypothesis for olfactory dysfunction based on DA-PGE2 interaction**Activating the dopamine D2 receptor by DA inhibits the release of inflammatory cytokines in the olfactory bulb microglia and protects olfactory function [29]. PGE2 can amplify both D1- and D2-receptor signaling through EP1. Dopamine receptor D2 agonists are potent in enhancing the release of arachidonic acid (AA) [20-24]. We propose that DA activity can antagonize PGE2 activity in the olfactory system and vice versa. Upregulated PGE2/COX-2 level is expected to decrease the activity of DA in tissues where EP1 is expressed [30,31]. All four receptors are expressed in the olfactory epithelium [32]. Thus, when the PGE2 level dominates the DA level, this could cause a loss of smell and taste. Pointing in that direction, in Parkinson's disease, a condition characterized by decreased DA levels, olfactory dysfunction is among the earliest nonmotor features [33]. As we mentioned above: Increased COX-2 is reported to cause oxidative protein modification and α-synuclein accumulation in dopaminergic cells [10], as seen in Parkinson’s disease [11-13].

**3. A hypothesis for post-exertional malaise (PEM) based on presumed DA-PGE2 interaction**Exercise is shown to upregulate DA [34, 35]. Exercise is shown to upregulate PGE2 as well [36, 37]. The latter seems to induce inflammation of the muscles in healthy subjects [36, 37].  We propose that exercise-derived DA could attenuate/postpone inflammation by antagonizing PGE2 activity. This assumption is based on research on the role of dopamine in exercise and inflammation [38] and the role of dopamine in endurance [39, 40] combined with the abovementioned published data (the bullet points). Inflammation of the muscles due to PGE2 upregulation could, under physiological conditions, be a way to prevent over-activity by forcing a halt to activity to save energy needed for recovery and survival. The baseline of PGE2 is higher in individuals already suffering from illness. In the case of a dominating PGE2 level, the DA raised by the exercise cannot compensate enough for the extra PGE2 raised by the same exercise. In that case, the inflammation process of the muscles is expected to start very soon after the beginning of the exercise. When PGE2 levels that are too high are already elevated by exercise, this could lead to post-exertional malaise (PEM). According to our hypothesis, a high level of PGE2 could induce resistance to substances that stimulate PGE2, causing extra inflammation, prolonging the recovery time, or even aggravating the symptoms experienced by many LC patients for weeks after exercise. 

**4. A hypothesis for orthostatic problems and tachycardia and bradycardia, based on presumed DA-PGE2 interaction**Long Covid patients may have orthostatic hypotension (OH1) as part of the POTS syndrome (Postural Orthostatic Tachycardia Syndrome) that is associated with impaired vagal and sympathetic cardiovascular control [41]. They may also experience orthostatic hypertension (OH2) after standing up or head-up tilt [42]. We propose this difference could be explained by ‘EP3 down’ or ‘EP3 up’ in the sympathetic nervous system. In those cases of unbalanced distribution, a rise of PGE2, following mental or physical stress could cause OH1 or OH2. A rise of dopamine (DA) could cause OH1 or OH2 as well, when the EP3 receptor is over- or under-expressed respectively.

How could a rise of PGE2-level cause OH1 and OH2?

Through the EP3-receptor PGE2 both antagonizes PNS activity and agonizes SNS activity (inflammatory). In case of over-availability of EP3 (EP3up): A rise of PGE2-level is expected to severely attenuate PNS activity, and at the same time to cause excessive SNS activity, increasing BP. When EP3 on the other hand, is insufficiently available (EP3 down), the PNS activity will dominate the SNS activity, leading to hypotension, especially in the upper part of the body, given that EP3 is also needed for the necessary vasoconstriction to prevent the blood from pooling in the lower part of the body (PGE2 is known to stimulate vasoconstriction through EP3).

Tachycardia and bradycardia: PGE2 decreases cardiac contractility through EP3 [43]. When EP3 is over-available a rise of PGE2 could lead to bradycardia. When EP3 is lacking, a rise of PGE2 could lead to tachycardia, especially when vasoconstriction is impaired and BP drops, inducing extra upregulation of PGE2.

How could a rise of DA-level cause OH1 and OH2?

Orthostatic symptoms are often induced by movement or stimulation of the senses. Dopamine (DA) plays a role in the vestibular organ [44-46]. It has been shown that DA activity is needed for keeping balance [47].  DA is needed for and stimulated by physical, emotional and cognitive activity and by using the senses [48]. OH1 and OH2 are also known to be induced by other circumstances that increase DA level (alcohol, nose spray).

Hormones are known to share subcellular signaling pathways. We propose that DA levels influence PGE2 signaling via the Gi-coupled EP3 receptor (see main article, section 5.1.4), antagonizing inflammatory actions mediated by EP3 while simultaneously agonizing other EP3-related functions through the Gi-coupled dopamine receptors D2, D3, and D4. This mechanism could explain dopamine’s anti-analgesic, anti-inflammatory, and body-temperature–lowering effects, as well as its stimulation of vasoconstriction at higher doses.
 We saw earlier that PGE2 attenuates PNS signaling through EP3. Thus, a rise in DA level can increase signaling in the PNS, stimulating lower BP. However, when DA is an EP3-agonist at the same time, it could stimulate (anti-inflammatory) SNS activity too. This way, a rise in DA could increase the signaling in the SNS, stimulating higher BP. Under physiological conditions, these increases in PNS and SNS signaling are expected to balance each others activity. But when EP3 is insufficiently available, the PNS action will dominate the SNS action, leading to hypotension, like in the case of PGE2, especially in the upper part of the body. On the other hand, when EP3 is over-available, DA upregulation is expected to lead to higher blood pressure, because the BP raising influence of the overactive SNS, will dominate the BP-lowering effect of the PNS. Of significance: As mentioned above, PGE2 and DA influence each other's activity via the EP1 receptor. Excess or insufficient EP1 expression could also substantially contribute to dysautonomia.

**References**

1.        Tsuge K, Inazumi T, Shimamoto A, Sugimoto Y. Molecular mechanisms underlying prostaglandin E2-exacerbated inflammation and immune diseases. Int Immunol. 2019;31(9):597-606. DOI: [10.1093/intimm/dxz021](https://doi.org/10.1093/intimm/dxz021)

2.        Moore SC, Vaz de Castro PAS, Yaqub D, Jose PA, Armando I. Anti-Inflammatory Effects of Peripheral Dopamine. Int J Mol Sci. 2023;24(18). DOI: [10.3390/ijms241813816](https://doi.org/10.3390/ijms241813816)

3.        Nakamura K, Katoh H, Ichikawa A, Negishi M. Inhibition of dopamine release by prostaglandin EP3 receptor via pertussis toxin-sensitive and -insensitive pathways in PC12 cells. J Neurochem. 1998;71(2):646-52. DOI: [10.1046/j.1471-4159.1998.71020646.x](https://doi.org/10.1046/j.1471-4159.1998.71020646.x)

4.        Bin NR, Prescott SL, Horio N, Wang Y, Chiu IM, Liberles SD. An airway-to-brain sensory pathway mediates influenza-induced sickness. Nature. 2023;615(7953):660-7.
 DOI: [10.1038/s41586-023-05796-0](https://doi.org/10.1038/s41586-023-05796-0) 5.        Fritz M, Klawonn AM, Nilsson A, Singh AK, Zajdel J, Björk Wilhelms D, et al. Prostaglandin-dependent modulation of dopaminergic neurotransmission elicits inflammation-induced aversion in mice. The Journal of Clinical Investigation. 2016;126(2):695-705. DOI: [10.1172/JCI83844](https://doi.org/10.1172/jci83844)

6.        Okazaki A, Hara J, Ohkura N, Fujimura M, Sakai T, Abo M, et al. Role of prostaglandin E(2) in bronchoconstriction-triggered cough response in guinea pigs. Pulm Pharmacol Ther. 2018;48:62-70. DOI: [10.1016/j.pupt.2017.09.003](https://doi.org/10.1016/j.pupt.2017.09.003)

7.        Zhou J, Alvarez-Elizondo MB, Botvinick E, George SC. Adenosine A(1) and prostaglandin E receptor 3 receptors mediate global airway contraction after local epithelial injury. Am J Respir Cell Mol Biol. 2013;48(3):299-305. DOI: [10.1165/rcmb.2012-0174OC](https://doi.org/10.1165/rcmb.2012-0174oc)

8.        Cabezas GA, Israili ZH, Velasco M. The actions of dopamine on the airways. Am J Ther. 2003;10(6):477-86. DOI: [10.1097/00045391-200311000-00019](https://doi.org/10.1097/00045391-200311000-00019)

9.        Carrasco E, Casper D, Werner P. PGE(2) receptor EP1 renders dopaminergic neurons selectively vulnerable to low-level oxidative stress and direct PGE(2) neurotoxicity. J  Neurosci Res. 2007;85(14):3109-17. DOI: [10.1002/jnr.21425](https://doi.org/10.1002/jnr.21425)

10.     Chae S-W, Kang BY, Hwang O, Choi HJ. Cyclooxygenase-2 is involved in oxidative damage and alpha-synuclein accumulation in dopaminergic cells. Neuroscience Letters. 2008;436(2):205-9. DOI: [10.1016/j.neulet.2008.03.031](https://doi.org/10.1016/j.neulet.2008.03.031)

11.     Yildirim C, Fenyi A, Besnault P, Gomez L, Sepulveda-Diaz JE, Michel PP, et al. Parkinson’s disease-derived α-Synuclein assemblies combined with chronic-type inflammatory cues promote a neurotoxic microglial phenotype. bioRxiv. 2023:2023.11.30.569208.
 DOI: [10.1186/s12974-024-03043-5](https://doi.org/10.1186/s12974-024-03043-5)

12.     Shin JY, Lee PH. Mesenchymal stem cells modulate misfolded α-synuclein in parkinsonian disorders: A multitarget disease-modifying strategy. Stem Cell Research. 2020;47:101908.
 DOI: [10.1016/j.scr.2020.101908](https://doi.org/10.1016/j.scr.2020.101908)

13.     Jin J, Shie FS, Liu J, Wang Y, Davis J, Schantz AM, et al. Prostaglandin E2 receptor subtype 2 (EP2) regulates microglial activation and associated neurotoxicity induced by aggregated alpha-synuclein. J Neuroinflammation. 2007;4:2. DOI: [10.1186/1742-2094-4-2](https://doi.org/10.1186/1742-2094-4-2)

14.     Tanaka Y, Furuyashiki T, Momiyama T, Namba H, Mizoguchi A, Mitsumori T, et al. Prostaglandin E receptor EP1 enhances GABA-mediated inhibition of dopaminergic neurons in the substantia nigra pars compacta and regulates dopamine level in the dorsal striatum. Eur J Neurosci. 2009;30(12):2338-46. DOI: [10.1111/j.1460-9568.2009.07021.x](https://doi.org/10.1111/j.1460-9568.2009.07021.x)

15.     Furuyashiki T, Narumiya S. Stress responses: the contribution of prostaglandin E(2) and its receptors. Nat Rev Endocrinol. 2011;7(3):163-75. DOI: [10.1038/nrendo.2010.194](https://doi.org/10.1038/nrendo.2010.194)

16.     Haddad A, Flint-Ashtamker G, Minzel W, Sood R, Rimon G, Barki-Harrington L. Prostaglandin EP1 receptor down-regulates expression of cyclooxygenase-2 by facilitating its proteasomal degradation. J Biol Chem. 2012;287(21):17214-23. DOI: [10.1074/jbc.M111.304220](https://doi.org/10.1074/jbc.m111.304220)

17.     Mbonye UR, Yuan C, Harris CE, Sidhu RS, Song I, Arakawa T, et al. Two Distinct Pathways for Cyclooxygenase-2 Protein Degradation*. Journal of Biological Chemistry. 2008;283(13):8611-23.

DOI: [10.1074/jbc.M710137200](https://doi.org/10.1074/jbc.m710137200)

18.     Matsuoka Y, Furuyashiki T, Yamada K, Nagai T, Bito H, Tanaka Y, et al. Prostaglandin E receptor EP1 controls impulsive behavior under stress. Proc Natl Acad Sci U S A. 2005;102(44):16066-71.<https://doi.org/10.1073/pnas.0504908102>

19.     Ehrlich AT, Furuyashiki T, Kitaoka S, Kakizuka A, Narumiya S. Prostaglandin E receptor EP1 forms a complex with dopamine D1 receptor and directs D1-induced cAMP production to adenylyl cyclase 7 through mobilizing G(βγ) subunits in human embryonic kidney 293T cells. Mol Pharmacol. 2013;84(3):476-86. DOI: [10.1124/mol.113.087288](https://doi.org/10.1124/mol.113.087288)

20.     Piomelli D, Di Marzo V. Dopamine D2 receptor signaling via the arachidonic acid cascade: modulation by cAMP-dependent protein kinase A and prostaglandin E2. J Lipid Mediat. 1993;6(1-3):433-43. PMID: **8395253**

21.     Nilsson CL, Hellstrand M, Ekman A, Eriksson E. Direct dopamine D2-receptor-mediated modulation of arachidonic acid release in transfected CHO cells without the concomitant administration of a Ca2+-mobilizing agent. Br J Pharmacol. 1998;124(8):1651-8.
 DOI: [10.1038/sj.bjp.0702025](https://doi.org/10.1038/sj.bjp.0702025)

22.     Hellstrand M, Eriksson E, Nilsson C. Dopamine D2 receptor-induced COX-2-mediated production of prostaglandin E2 in D2-transfected Chinese hamster ovary cells without simultaneous administration of a Ca2+-mobilizing agent. Biochemical pharmacology. 2002;63:2151-8.
 DOI: [10.1016/s0006-2952(02)01020-1](https://doi.org/10.1016/s0006-2952(02)01020-1)

23.     Di Marzo V, Piomelli D. Participation of Prostaglandin E2 in Dopamine D2 Receptor-Dependent Potentiation of Arachidonic Acid Release. Journal of Neurochemistry. 1992;59(1):379-82.

DOI: [10.1111/j.1471-4159.1992.tb08915.x](https://doi.org/10.1111/j.1471-4159.1992.tb08915.x)

24.     Kitaoka S, Furuyashiki T, Nishi A, Shuto T, Koyasu S, Matsuoka T, et al. Prostaglandin E2 acts on EP1 receptor and amplifies both dopamine D1 and D2 receptor signaling in the striatum. J Neurosci. 2007;27(47):12900-7. DOI: [10.1523/JNEUROSCI.3257-07.2007](https://doi.org/10.1523/jneurosci.3257-07.2007)

25.     Wang B, Wu L, Chen J, Dong L, Chen C, Wen Z, et al. Metabolism pathways of arachidonic acids: mechanisms and potential therapeutic targets. Signal Transduct Target Ther. 2021;6(1):94.25. DOI: [10.1038/s41392-020-00443-w](https://doi.org/10.1038/s41392-020-00443-w)

26.     Bonilla H, Peluso MJ, Rodgers K, Aberg JA, Patterson TF, Tamburro R, et al. Therapeutic trials for long COVID-19: A call to action from the interventions taskforce of the RECOVER initiative. Front Immunol. 2023;14:1129459. DOI: [10.3389/fimmu.2023.1129459](https://doi.org/10.3389/fimmu.2023.1129459)

27.     Li Z, Ichikawa J, Dai J, Meltzer HY. Aripiprazole, a novel antipsychotic drug, preferentially increases dopamine release in the prefrontal cortex and hippocampus in rat brain. European Journal of Pharmacology. 2004;493(1):75-83. DOI: [10.1016/j.ejphar.2004.04.028](https://doi.org/10.1016/j.ejphar.2004.04.028)

28.     Jordan S, Johnson JL, Regardie K, Chen R, Koprivica V, Tadori Y, et al. Dopamine D2 receptor partial agonists display differential or contrasting characteristics in membrane and cell-based assays of dopamine D2 receptor signaling. Prog Neuropsychopha rmacol Biol Psychiatry. 2007;31(2):348-56.

DOI: [10.1016/j.pnpbp.2006.09.007](https://doi.org/10.1016/j.pnpbp.2006.09.007)

29.     Liu P, Qin D, Lv H, Fan W, Zhou F, Gao Z, et al. Activation of Dopamine D2 Receptor Alleviates Neuroinflammation in a Mouse Model of Allergic Rhinitis With Olfactory Dysfunction. Allergy Asthma Immunol Res. 2021;13(6):882-95. DOI: [10.4168/aair.2021.13.6.882](https://doi.org/10.4168/aair.2021.13.6.882)

30.     Suzuki C, Miyamoto C, Furuyashiki T, Narumiya S, Ohinata K. Central PGE2 exhibits anxiolytic-like activity via EP1 and EP4 receptors in a manner dependent on serotonin 5-HT1A, dopamine D1 and GABAA receptors. FEBS Letters. 2011;585(14):2357-62. DOI: [10.1016/j.febslet.2011.06.004](https://doi.org/10.1016/j.febslet.2011.06.004)

31.     Ahmad AS, Maruyama T, Narumiya S, Doré S. PGE2 EP1 receptor deletion attenuates 6-OHDA-induced Parkinsonism in mice: old switch, new target. Neurotox Res. 2013;23(3):260-6.
 DOI: [10.1007/s12640-013-9381-8](https://doi.org/10.1007/s12640-013-9381-8)

32.     Fukuiri T, Takumida M, Nakashimo Y, Hirakawa K. Expression of prostanoid receptors (EP1, 2, 3, and 4) in normal and methimazole-treated mouse olfactory epithelium. Acta Otolaryngol. 2013;133(1):70-6. DOI: [10.3109/00016489.2012.712214](https://doi.org/10.3109/00016489.2012.712214)

33.     Fullard ME, Morley JF, Duda JE. Olfactory Dysfunction as an Early Biomarker in Parkinson's Disease. Neurosci Bull. 2017;33(5):515-25. DOI: [10.1007/s12264-017-0170-x](https://doi.org/10.1007/s12264-017-0170-x)

34.     J. Tyler, M. Podaras, B. Richardson, N. Roeder, N. Hammond, J. Hamilton, et al. High intensity interval training exercise increases dopamine D2 levels and modulates brain dopamine signaling. Front Public Health 2023 Vol. 11 Pages 1257629.<https://doi.org/10.3389/fpubh.2023.1257629>
35.     Guendalina Bastioli, Jennifer C. Arnold, Maria Mancini, Adam C. Mar, Margaret E. Rice et al. Voluntary Exercise Boosts Striatal Dopamine Release: Evidence for the Necessary and Sufficient Role of BDNF. Journal of Neuroscience 8 June 2022, 42 (23) 4725-4736;<https://doi.org/10.1523/JNEUROSCI.2273-21.2022>
36.     I. Okayasu, H. Kuroiwa, K. Shinkawa, K. Hayashi, S. Sato, N. Iwata, et al. Significant increase in prostaglandin E-major urinary metabolite with physical exercise suggesting muscle inflammation. All Life 2023 Vol. 16 Issue 1 Pages 2167868.<https://doi.org/10.1080/26895293.2023.2167868>
37. Lavin KM, Perkins RK, Jemiolo B, Raue U, Trappe SW, Trappe TA. Effects of aging and lifelong aerobic exercise on basal and exercise-induced inflammation. J Appl Physiol (1985). 2020 Jan 1;128(1):87-99. DOI: [10.1152/japplphysiol.00495.2019](https://doi.org/10.1152/japplphysiol.00495.2019)

38.     F. S. Lira, A. S. Yamashita, J. C. Rosa, C. H. Koyama, E. C. Caperuto, M. L. Batista, Jr., et al. Exercise training decreases adipose tissue inflammation in cachectic rats. Horm Metab Res 2012 Vol. 44 Issue 2 Pages 91-8. DOI: [10.1055/s-0031-1299694](https://doi.org/10.1055/s-0031-1299694)
39.     Zheng X, Hasegawa H. Central dopaminergic neurotransmission plays an important role in thermoregulation and performance during endurance exercise.
 DOI: [10.1080/17461391.2015.1111938](https://doi.org/10.1080/17461391.2015.1111938)
40.     Meeusen R, Roelands B. Central fatigue and neurotransmitters, can thermoregulation be manipulated? Scand J Med Sci Sports. 2010 Oct;20 Suppl 3:19-28.
 DOI: [10.1111/j.1600-0838.2010.01205.x](https://doi.org/10.1111/j.1600-0838.2010.01205.x)
41.     Jacob G, Diedrich L, Sato K, Brychta RJ, Raj SR, Robertson D, et al. Vagal and Sympathetic Function in Neuropathic Postural Tachycardia Syndrome. Hypertension. 2019;73(5):1087-96.
 DOI: [10.1161/HYPERTENSIONAHA.118.11803](https://doi.org/10.1161/hypertensionaha.118.11803)
42.     Goodman BP, Khoury JA, Blair JE, Grill MF. COVID-19 Dysautonomia. Front Neurol. 2021;12:624968. DOI: [10.3389/fneur.2021.624968](https://doi.org/10.3389/fneur.2021.624968)

43.     Gu X, Xu J, Zhu L, Bryson T, Yang XP, Peterson E, et al. Prostaglandin E2 Reduces Cardiac Contractility via EP3 Receptor. Circ Heart Fail. 2016;9(8).
 DOI: [10.1161/CIRCHEARTFAILURE.116.003291](https://doi.org/10.1161/circheartfailure.116.003291)
44.     Canton-Josh JE, Qin J, Salvo J, Kozorovitskiy Y. Dopaminergic regulation of vestibulo-cerebellar circuits through unipolar brush cells. eLife. 2022;11:e76912.
DOI: [10.7554/eLife.76912](https://doi.org/10.7554/elife.76912)
45.     Oestreicher E, Arnold W, Ehrenberger K, Felix D. Dopamine regulates the glutamatergic inner hair cell activity in guinea pigs. Hearing Research. 1997;107(1):46-52.
 DOI: [10.1016/s0378-5955(97)00023-3](https://doi.org/10.1016/s0378-5955(97)00023-3)
46. Toro C, Trapani JG, Pacentine I, Maeda R, Sheets L, Mo W, et al. Dopamine Modulates the Activity of Sensory Hair Cells. J Neurosci. 2015;35(50):16494-503.
 DOI: [10.1523/JNEUROSCI.1691-15.2015](https://doi.org/10.1523/jneurosci.1691-15.2015)
47.     Jansen N, Feuerecker R, Dieterich M, Bartenstein P, la Fougere C. Assessment of extrastriatal dopamine D2-receptors in patients with bilateral vestibular failure. Journal of Nuclear Medicine. 2012;53(supplement 1):1944-. DOI: [10.3233/VES-140526](https://doi.org/10.3233/ves-140526)
48.     Wall VZ, Parker JG, Fadok JP, Darvas M, Zweifel L, Palmiter RD. A behavioral genetics approach to understanding D1 receptor involvement in phasic dopamine signaling. Mol Cell Neurosci. 2011;46(1):21-31. DOI: [10.1016/j.mcn.2010.09.011](https://doi.org/10.1016/j.mcn.2010.09.011)

(48)
